# Atrophic Skeletal Muscle‐Derived Extracellular Vesicles Transfer miR‐125a‐5p to Inhibit Bone Formation in Osteoporosis during Aging

**DOI:** 10.1002/advs.202515362

**Published:** 2026-02-26

**Authors:** Xiaoyan Shao, Pan Zhang, Zhidan Fan, Jiaquan Lin, Xiang Chen, Na Liu, Wang Gong, Yi He, Yining Zhou, Tianshu Shi, Yong Shi, Yuze Ma, Wentian Gao, Haosheng Wang, Depeng Fang, Chengzhi Wang, Wenshu Wu, Wenjin Yan, Jianghui Qin, Dongyang Chen, Haiguo Yu, Qing Jiang, Baosheng Guo

**Affiliations:** ^1^ Division of Sports Medicine and Adult Reconstructive Surgery Department of Orthopedic Surgery Nanjing Drum Tower Hospital State Key Laboratory of Pharmaceutical Biotechnology Nanjing University Nanjing Jiangsu P. R. China; ^2^ Department of Rheumatology and Immunology Children's Hospital of Nanjing Medical University Nanjing P. R. China; ^3^ Branch of National Clinical Research Center for Orthopedics Sports Medicine and Rehabilitation Nanjing P. R. China; ^4^ Jiangsu Key Laboratory of Molecular Medicine Medical School Nanjing University Nanjing Jiangsu P. R. China; ^5^ State Key Laboratory of Pharmaceutical Biotechnology Nanjing University Nanjing Jiangsu P. R. China; ^6^ Department of Orthopedics The First Affiliated Hospital of Wenzhou Medical University Wenzhou Zhejiang P. R. China

**Keywords:** bone formation, extracellular vesicles, MiR‐125a‐5p, muscle atrophy, osteoporosis

## Abstract

Understanding how skeletal muscle influences bone formation is essential for uncovering the mechanisms of muscle‐bone communication and developing therapies for osteoporosis. Here, we demonstrate that extracellular vesicles (EVs) derived from atrophic skeletal muscle (Aged‐SKM‐EVs) inhibit bone formation during aging. Utilizing a muscle‐specific EV tracking transgenic mouse model, we found that Aged‐SKM‐EVs were significantly increased and taken up by osteoblasts in bone during aging. Notably, pharmacological blockade of muscle EV generation via a skeletal muscle‐targeted delivery of GW4869 significantly restored osteoblast activity and alleviated bone loss in aged mice. Functional studies revealed that Aged‐SKM‐EVs suppressed bone formation and inhibited osteogenic differentiation both in vivo and in vitro. Mechanistically, we identified miR‐125a‐5p as a key cargo enriched in EVs from sarcopenic patients and aged mice. Muscle‐specific overexpression of miR‐125a‐5p inhibited osteogenesis and exacerbated muscle atrophy and bone loss, whereas silencing miR‐125a‐5p in skeletal muscle effectively reversed these effects. Further investigation demonstrated that miR‐125a‐5p inhibits osteogenic differentiation by directly targeting *Sirt7* in preosteoblasts, thereby disrupting SIRT7‐mediated histone deacetylation at the *Sp7* promoter and suppressing *Sp7* transcription. Our findings reveal a novel endocrine pathway from muscle to bone mediated by EV‐associated miRNA and highlight miR‐125a‐5p as a promising therapeutic target for sarcopenia‐related osteoporosis.

## Graphical abstract

A muscle‐bone endocrine pathway in aging is revealed in which extracellular vesicles released from atrophic skeletal muscle (Aged‐SKM‐EVs) inhibit bone formation. These EVs deliver miR‐125a‐5p to osteoblasts, thereby suppressing the SIRT7‐*Sp7* signaling axis and osteogenic differentiation. Therapeutic targeting of Aged‐SKM‐EVs or miR‐125a‐5p alleviates sarcopenia‐associated bone loss.

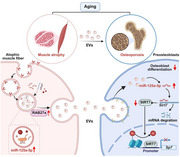

## 1 Introduction

Skeletal muscle and bone, the two principal components of the musculoskeletal system, exhibit a highly coordinated relationship from development through aging [1]. During aging, muscle atrophy and osteoporosis frequently co‐occur in the same individuals, thereby compromising mobility and quality of life, and significantly increasing the risk of fractures and other serious health complications [2]. Importantly, under pathological conditions, skeletal muscle atrophy often precedes and contributes to bone loss. Numerous studies have demonstrated that individuals with sarcopenia are at an elevated risk of developing senile osteoporosis [3]. However, the cellular and molecular mechanisms underlying senile osteoporosis remain inadequately understood. Consequently, elucidating the molecular communication between skeletal muscle and bone is crucial for developing effective strategies to prevent osteoporosis in the elderly.

Skeletal muscle functions as an endocrine organ, secreting a variety of myokines that regulate the metabolism of other tissues [4]. Recently, an increasing number of myokines have been identified as regulators of bone metabolism [5]. For example, myostatin, irisin, IGF‐1, FGF2, decorin, and IL‐6 have been identified as positive regulators of bone formation, whereas myostatin and FGF21 act as negative regulators of bone resorption [6]. Beyond these conventional paracrine factors, skeletal muscle is also capable of secreting extracellular vesicles (EVs), which have been implicated in critical inter‐organ communication and the regulation of recipient organ functions [7, 8, 9]. Research has demonstrated that EVs derived from C2C12 myoblasts facilitate the osteogenic differentiation of pre‐osteoblastic MC3T3‐E1 cells into mature osteoblasts by activating the β‐catenin signaling pathway [10]. Furthermore, several studies indicate that pathological conditions, such as injury, atrophy, and aging, can modify the cargo of muscle‐derived EVs [11, 12]. For instance, aging‐related oxidative stress in skeletal muscle is associated with an increased expression of the senescence‐associated microRNA, miR‐34a, in both skeletal muscle and muscle‐derived circulating EVs, which has been shown to induce senescence in bone marrow stromal cells (BMSCs) [8]. These findings suggest that EVs derived from skeletal muscle may play an important role in muscle‐bone crosstalk during aging. However, the underlying mechanisms remain to be elucidated.

In this study, we demonstrate that atrophic skeletal muscle‐derived EVs (Aged‐SKM‐EVs) are transferred to bone tissue and internalized by osteoblasts during aging. We further demonstrated that these Aged‐SKM‐EVs inhibit osteogenic differentiation in vitro and suppress bone formation in vivo. Mechanistically, we found that miR‐125a‐5p is significantly upregulated in plasma EVs of elderly patients with sarcopenia (SAR‐EVs), as well as in Aged‐SKM‐EVs and atrophic myotubes. Moreover, miR‐125a‐5p was shown to inhibit osteogenic differentiation by directly targeting *Sirt7* in preosteoblasts. SIRT7 was directly associated with the *Sp7* promoter, where it facilitated histone deacetylation and enhanced *Sp7* transcription. Thus, miR‐125a‐5p mediated suppression of *Sirt7* impaired *Sp7* transcriptional activity and ultimately attenuated osteoblast differentiation. Collectively, these findings elucidate a novel mechanism by which Aged‐SKM‐EVs enriched in miR‐125a‐5p suppress bone formation, thereby revealing a previously unrecognized muscle‐bone communication axis during aging.

## 2 Results

### 2.1 Atrophic Skeletal Muscle‐Derived EVs are Increased and Taken up by Osteoblasts in Bone during Aging‐Induced Muscle Atrophy

**FIGURE 1:**
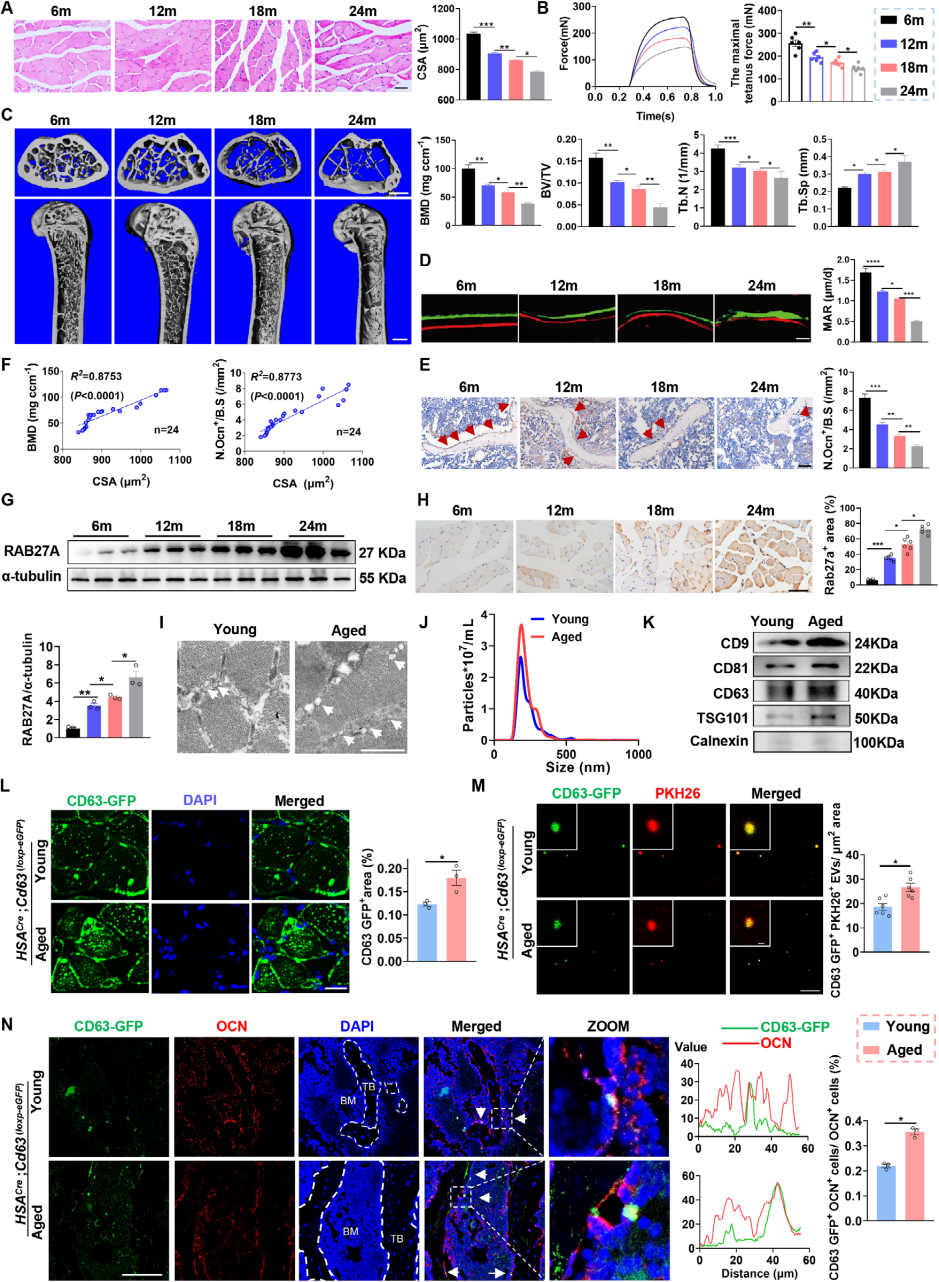
Aged‐SKM‐EVs are increased and taken up by osteoblasts in bone during aging. (A) Representative Hematoxylin and Eosin (H&E) staining of cross‐sections from the mid‐belly of soleus (SOL) muscles and quantification of muscle fiber cross‐sectional area (CSA). *n = 6*. Scale bar, 100 µm. (B) Representative tetanic force curve of SOL muscles from 6‐, 12‐, 18‐, and 24‐month‐old mice and quantification of maximal specific force during tetanic stimulation. *n = 6*. (C) Representative micro‐CT reconstructed images and quantitative analysis of bone mineral density (BMD), bone volume/total volume (BV/TV), trabecular number (Tb.N), and trabecular separation (Tb.Sp) at the distal femoral metaphysis. Scale bar, 100 µm (upper panels) and 500 µm (lower panels). *n = 6*. (D) Representative images of double fluorochrome labeling with calcein green and xylenol orange at the distal femoral metaphysis and quantitative bone histomorphometric analysis of mineral apposition rate (MAR). Scale bar, 25 µm. *n = 6*. (E) Representative immunohistochemical staining of osteocalcin (OCN) in distal femoral sections and quantification of osteoblast surface per bone surface (Ob.S/BS). Scale bar, 50 µm. *n = 6*. (F) Individual correlations between SOL muscle fiber CSA and BMD, and between CSA and Ob.S/BS. *n = 24*. (G) Western blot analysis and quantification of protein expression of RAB27A expression in SOL muscles. *n = 3*. (H) Representative immunohistochemical staining and quantification of RAB27A‐positive areas in SOL muscles. Scale bar, 100 µm. *n = 6*. (I) Representative transmission electron microscopy (TEM) images of SKM‐EVs. Scale bar, 1 µm. (J) Nanoparticle tracking analysis (NTA) showing particle size distribution and concentration of SKM‐EVs. (K) Western blot analysis of canonical EV markers in SKM‐EVs. (L) Distribution and quantification of eGFP‐labeled EVs in skeletal muscle from *HSA^Cre^* *;Cd63* ^(^ *^loxp^* ^−^ *^eGFP^* ^)^ mice. Scale bar, 100 µm. *n = 3*. (M) Representative fluorescence images showing circulating eGFP^+^PKH26^+^ EVs in plasma from *HSA^Cre^;Cd63 ^(loxp‐eGFP)^* mice, and quantification of the number density of eGFP^+^PKH26^+^ EVs (per µm^2^). Scale bar, 100 nm. *n = 6*. (N) Representative immunofluorescence staining of OCN in femoral sections (left), quantitative colocalization analysis (middle), and quantification of the percentage of CD63 GFP^+^ OCN^+^ cells among total OCN^+^ cells (right). Nuclei were co‐stained with DAPI. Scale bar, 100 µm. *n = 3*. All data are presented as mean ± SEM. *P* values were determined by one‐way analysis of variance (ANOVA) followed by Tukey's multiple comparisons test (A–E, G–H), or unpaired two‐tailed Student's t test (L–N). ^*^P< 0.05, ^**^P< 0.01, ^***^P< 0.001.

Senile osteoporosis frequently co‐occurs with sarcopenia during aging [13] and is mainly due to impaired osteoblastic bone formation [13, 14, 15]. Given the release of Aged‐SKM‐EVs during aging [7, 8, 9], our study aimed to investigate whether these EVs could be taken up by osteoblasts. Consistent with previous studies [7, 16], we observed a progressive decline in muscle fiber cross‐sectional area (CSA) and contractile function in both the soleus (SOL) (Figure 1A,B) and extensor digitorum longus (EDL) muscles (Figure S1A,B) in mice aged 6, 12, 18, and 24 months. These changes were accompanied by increased muscle fibrosis (Figure S1C), reduced bone mass (Figure 1C), and impaired osteoblastic bone formation at the distal femoral metaphysis (Figure S1D,E). Notably, muscle fiber CSA showed a significant positive correlation with both bone mineral density (BMD) and the ratio of osteoblast surface to bone surface (Ob.S/BS) across different ages (Figure 1F), indicating a close association between muscle atrophy and bone loss during aging.

Considering the pivotal role of RAB27A in facilitating the docking of multivesicular bodies at the plasma membrane during exosome secretion, thereby serving as a marker of exosome release [17], we investigated RAB27A expression in muscle fibers from mice aged 6, 12, 18, and 24 months. Notably, both western blot analysis (Figure 1G) and immunohistochemical staining (Figure 1H) revealed a significant increase in RAB27A protein expression in muscle fibers undergoing aging‐induced atrophy. Furthermore, transmission electron micrographs (TEM) showed an increased number of intraluminal vesicles (ILVs) in the muscle tissue of aged mice compared to young mice (Figure 1I). Subsequently, we isolated EVs from the muscle tissues of both young and aged mice for further analysis. Nanoparticle tracking analysis (NTA) revealed a significantly higher abundance of EVs released from aged muscle tissues compared to those from young muscle tissues (Figure 1J). Western blot analysis confirmed that these EVs expressed classical EV markers, including CD9, CD63, CD81, and TSG101 (Figure 1K), with significantly higher expression levels in EVs derived from aged muscle compared with those from young muscle. Collectively, these findings suggest that atrophic skeletal muscle exhibits increased EV secretion during aging.

To further investigate whether muscle fiber‐specific EVs are taken up by osteoblasts in bone tissue during aging, we crossed *Cd63* ^(^ *^loxp^* ^−^ *^eGFP^* ^)^ exosome reporter mice with transgenic mice expressing Cre recombinase under the control of the human skeletal muscle actin (Hsa) promoter [18], successfully generating *HSA^Cre^*; *Cd63* ^(^ *^loxp^* ^−^ *^eGFP^* ^)^ mice (Figure S2A). This genetic modification enabled the transcription of eGFP in muscle fibers following the deletion of the floxed stop sequence by Cre recombinase. We observed stronger eGFP fluorescence in the muscle fibers of aged *HSA^Cre^; Cd63 ^(loxp‐eGFP)^* mice compared with young counterparts (Figure 1L). Consistently, plasma EVs were collected and labeled with PKH26, revealing a greater presence of eGFP fluorescent signals in plasma EVs from aged *HSA^Cre^; Cd63 ^(loxp‐eGFP)^* mice compared to those from young *HSA^Cre^; Cd63 ^(loxp‐eGFP)^* mice (Figure 1M). Importantly, eGFP‐positive signals were readily detected in osteoblasts within femoral bone sections, with an increase observed in aged *HSA^Cre^; Cd63 ^(loxp‐eGFP)^* mice (Figure 1N). Taken together, these results suggest that Aged‐SKM‐EVs are increased during aging and are taken up by osteoblasts in bone tissue. Accordingly, we next investigated the role of Aged‐SKM‐EVs in regulating osteoblast function during aging.

### 2.2 Specific Blockade of EV Generation in Skeletal Muscle by GW4869 Alleviates Bone Loss in Aged Mice

To investigate whether muscle fiber‐specific inhibition of EV generation influences bone formation in aged mice, we developed RGDLTTP peptide‐modified liposomes (R‐LS) to selectively target skeletal muscle fibers, as this peptide has been reported to exhibit high affinity for muscle tissue [19]. GW4869 was encapsulated within these liposomes (R‐LS‐GW4869) to selectively inhibit EV generation in skeletal muscle fibers (Figure S3A). Representative TEM images of R‐LS‐GW4869 (Figure S3B) reveal a typical spherical morphology, with an average particle size of approximately 170 nm (Figure S3C). IVIS Spectrum imaging demonstrated strong fluorescent signals in skeletal muscle following intravenous injection of DiR‐labeled R‐LS (DiR‐R‐LS), whereas minimal signals were detected in mice injected with PBS, free DiR, or DiR‐LS (Figure S3D). In addition, fluorescence signals in skeletal muscle were significantly higher in the DiR‐R‐LS–injected group, whereas signals in the heart, liver, spleen, lung, and kidney were significantly lower than those in the DiR and DiR‐LS groups (Figure S3E), indicating that R‐LS exhibits high in vivo selectivity for skeletal muscle fibers. These results suggest that R‐LS serves as an efficient and selective carrier for the targeted delivery of GW4869 to skeletal muscle fibers.

**FIGURE 2:**
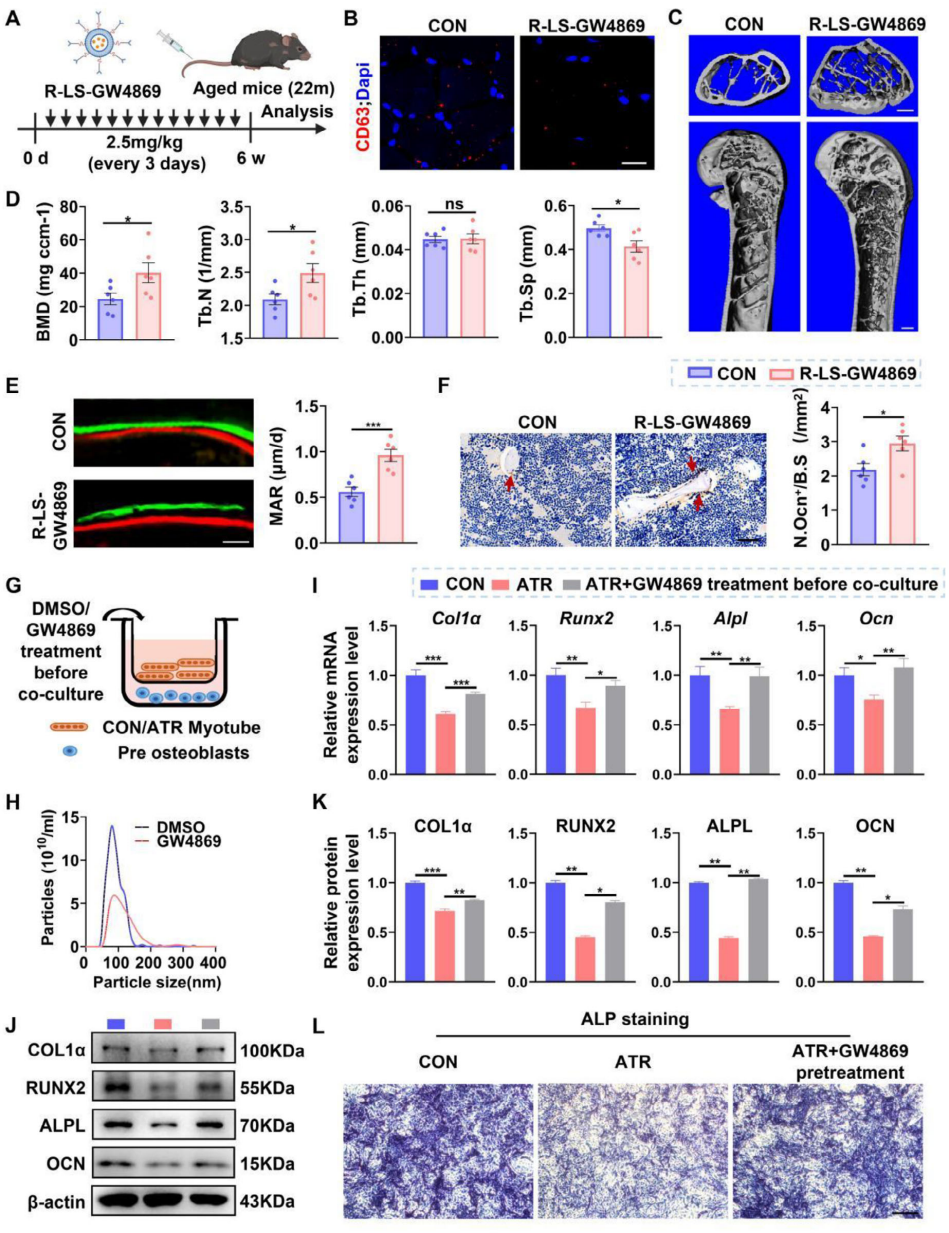
Muscle‐specific inhibition of EV generation alleviates bone loss in aged mice. (A) Schematic diagram illustrating the experimental design. Aged mice were intravenously injected with RGDLTTP peptide‐modified liposome capsulating GW4869 every three days for 6 weeks. (B) Representative immunofluorescent staining for CD63 in TA muscle sections. Nuclei were co‐stained with DAPI. Scale bars, 100 µm. (C) Representative micro‐CT reconstructed images of femora. Scale bar, 100 µm (upper panels) and 500 µm (lower panels). (D) Micro‐CT analysis of BMD, Tb.N, Tb.Th and Tb.Sp at the distal femoral metaphysis. *n = 6*. (E) Representative images of double fluorochrome labeling with calcein green and xylenol orange at the distal femoral metaphysis and quantitative bone histomorphometric analysis of MAR. Scale bar, 25 µm. *n = 6*. (F) Representative immunohistochemical staining of OCN in distal femoral sections and quantification of Ob.S/BS. Scale bar, 50 µm. *n = 6*. (G) Schematic diagram illustrating the experimental design of co‐culture preosteoblasts with control myotubes (CON), atrophic myotubes (ATR) or GW4869 pretreated atrophic myotubes (ATR+GW4869). Myotubes were treated with or without GW4869 (20 µм) before co‐culture. (H) NTA analysis of EVs derived from ATR myotubes treated with DMSO or GW4869. (I) qPCR analysis of osteogenic differentiation‐related genes in preosteoblast after co‐cultured with indicated myotubes. *n = 3*. (J,K) Western blot analysis and quantification of osteogenic differentiation‐related protein expression in osteoblasts after co‐cultured with indicated myotubes. *n = 3*. (L) Alkaline phosphatase (ALP) staining of osteoblasts after co‐cultured with indicated myotubes for 7 days, respectively. Scale bars, 500um. All data are presented as mean ± SEM. *P* values were determined by unpaired two‐tailed Student's *t* test (D‐F), or one‐way ANOVA followed by Tukey's multiple comparisons test (I, K). ^*^P< 0.05, ^**^P< 0.01, ^***^P< 0.001.

We next administered R‐LS‐GW4869 to aged mice via intravenous injection (i.v., 2.5 mg/kg) every 3 days for 6 weeks (Figure 2A) [20]. Treatment with R‐LS‐GW4869 significantly reduced muscle‐derived EVs (Figure 2B) and significantly suppressed nSMase2 expression in skeletal muscle (Figure S3F). Importantly, no significant changes in nSMase2 expression were observed in other organs compared with the control group (Figure S3F). Micro‐computed tomography (CT) analysis revealed improved bone architecture, higher BMD and trabecular number (Tb.N), along with reduced trabecular separation (Tb.Sp) in R‐LS‐GW4869–treated mice compared with PBS‐treated controls (Figure 2C,D). Calcein double labeling showed an increased mineral apposition rate (MAR) (Figure 2E), and immunohistochemical staining for OCN indicated a greater area of OCN positivity and higher Ob.S/BS at the distal femur (Figure 2F). Together, these results suggest that muscle‐specific inhibition of EV biogenesis by GW4869 promotes bone formation and alleviates age‐related bone loss in vivo.

To further investigate the effect in vitro, we established a starvation‐induced myotube atrophy model (Figure S4A,B). Preosteoblasts were co‐cultured with control myotubes, atrophic myotubes, or GW4869‐pretreated atrophic myotubes (Figure 2G). NTA analysis confirmed a reduction in EV number in the GW4869‐pretreated group (Figure 2H). Co‐culture with ATR myotubes significantly suppressed mRNA (Figure 2I) and protein (Figure S2J,K) expression of osteogenic genes, including *Collagen1α1*, *Runx2*, *Alpl*, and *Ocn*, in preosteoblasts. Notably, the inhibitory effect on osteogenic differentiation was reversed by GW4869 treatment. Consistently, alkaline phosphatase (ALP) staining showed decreased ALP activity in preosteoblasts co‐cultured with ATR myotubes, which was restored by GW4869 (Figure 2L). These findings indicate that blockade of EV biogenesis in atrophic myotubes alleviates their inhibitory effect on osteogenic differentiation in vitro.

### 2.3 Aged‐SKM‐EVs Inhibit Bone Formation and Osteogenic Differentiation In Vivo and In Vitro

**FIGURE 3:**
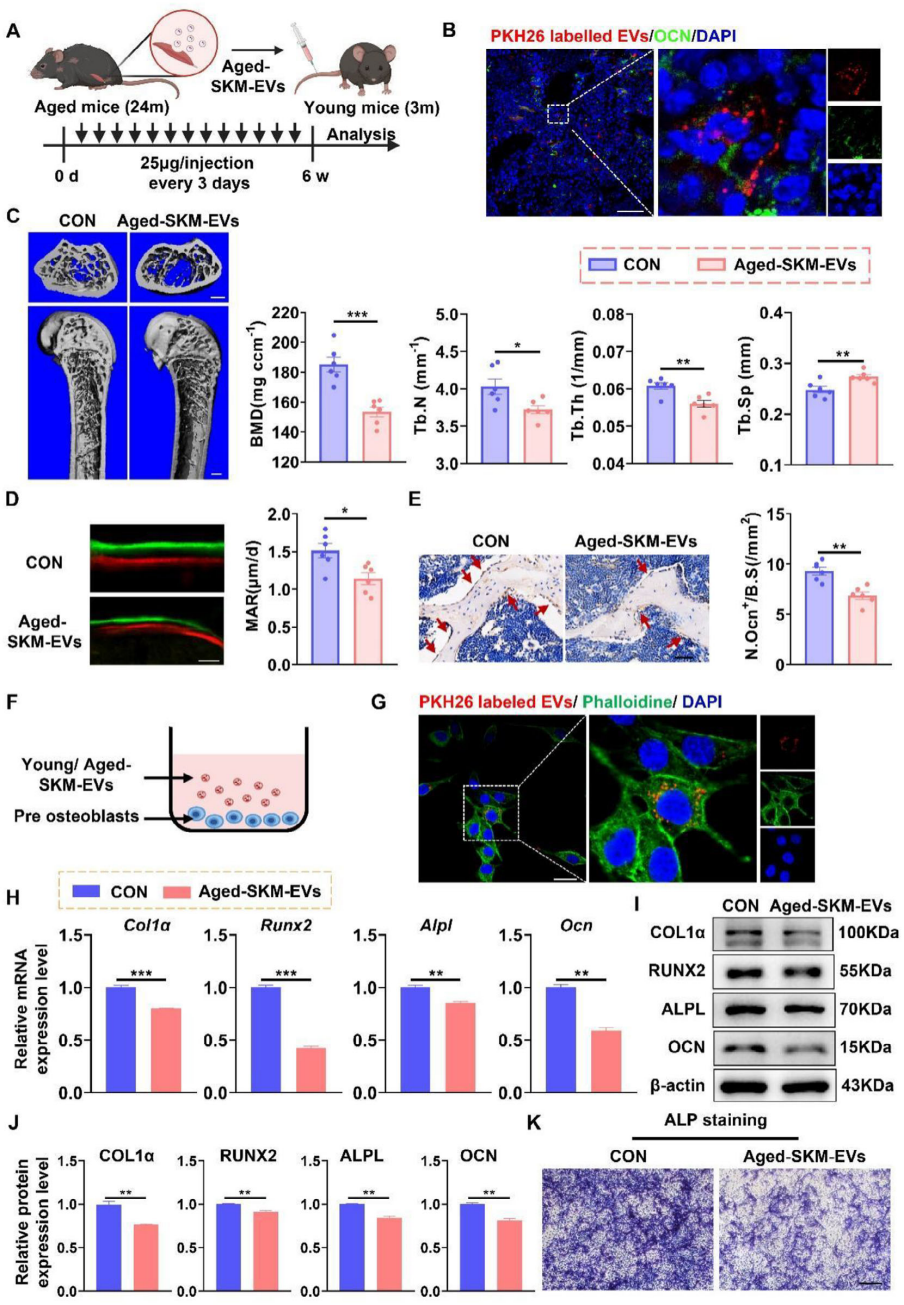
Aged‐SKM‐EVs inhibit bone formation and osteogenic differentiation in vivo and in vitro. (A) Schematic illustration of the experimental design. Young mice were intravenously injected with Aged‐SKM‐EVs every three days for 4 weeks. (B) Representative immunofluorescent staining for PKH26 and OCN in Femur sections of young mice 24 h after intramuscular injection with PKH26‐labeled Aged‐SKM‐EVs. Nuclei were co‐stained with DAPI (blue). Scale bars, 50 µm. (C) Representative micro‐CT reconstructed images and quantitative analysis of BMD, Tb.N, Tb.Th and Tb.Sp at the distal femoral metaphysis. Scale bar, 100 µm (upper panels) and 500 µm (lower panels). *n = 6*. (D) Representative images of double fluorochrome labeling with calcein green and xylenol orange at the distal femoral metaphysis and quantitative bone histomorphometric analysis of MAR. Scale bar, 25 µm. *n = 6*. (E) Representative immunohistochemical staining of OCN in distal femoral sections and quantification of Ob.S/BS. Scale bar, 50 µm. *n = 6*. (F) Schematic diagram illustrating the experimental design of preosteoblasts incubated with young SKM‐EVs or Aged‐SKM‐EVs. (G) Representative immunofluorescent staining for PKH26 and phalloidine in preosteoblasts after 24 h of incubation with PKH26‐labeled SKM‐EVs. Nuclei were co‐stained with DAPI (blue). Scale bars, 25 µm. (H) qPCR analysis of osteogenic differentiation‐related genes in preosteoblasts after incubated with young SKM‐EVs or Aged‐SKM‐EVs, respectively. *n = 3*. (I,J) Western blot analysis and quantification of osteogenic differentiation‐related protein expression in preosteoblasts after incubated with young SKM‐EVs or Aged‐SKM‐EVs, respectively. *n = 3*. (K) ALP staining of osteoblasts after incubated with young SKM‐EVs or Aged‐SKM‐EVs for 7 days, respectively. Scale bars, 500um. All data are presented as mean ± SEM. *P* values were determined by unpaired two‐tailed Student's *t* test (C‐E, H, J). ^*^P< 0.05, ^**^P< 0.01, ^***^P< 0.001.

To investigate whether the Aged‐SKM‐EVs influence bone formation in vivo, young mice were intravenously injected with Aged‐SKM‐EVs every 3 days for 6 weeks (Figure 3A). PKH26‐labeled Aged‐SKM‐EVs were efficiently internalized by osteoblasts in the femur of young mice (Figure 3B). Micro‐CT analysis revealed decreased BMD, Tb.N, and Tb.Th, along with increased Tb.Sp, at the distal femur in mice administered Aged‐SKM‐EVs compared with PBS‐treated controls (Figure 3C). Calcein double labeling showed a reduced MAR (Figure 3D), and immunohistochemical staining for OCN indicated decreased OCN‐positive area and lower Ob.S/BS at the distal femur (Figure 3E). Collectively, these results indicate that Aged‐SKM‐EVs directly inhibit bone formation in vivo. To evaluate the effects of Aged‐SKM‐EVs on osteogenic differentiation in vitro, preosteoblasts were cultured with Aged‐SKM‐EVs or SKM‐EVs from young mice (Figure 3F). PKH26‐labeled SKM‐EVs were efficiently internalized by preosteoblasts (Figure 3G). Incubation with Aged‐SKM‐EVs significantly suppressed mRNA (Figure 3H) and protein (Figure 3I,J) expression of osteogenic genes, including *Collagen1α1*, *Runx2*, *Alpl*, and *Ocn*, compared with the control group. Consistently, ALP staining revealed decreased ALP activity in preosteoblasts treated with Aged‐SKM‐EVs (Figure 3K).

We further assessed the inhibitory effects of atrophic myotube‐derived EVs (ATR‐EVs) on osteogenic differentiation in vitro by treating preosteoblastic MC3T3‐E1 cells with ATR‐EVs or control myotube‐derived EVs (CON‐EVs) (Figure S5A). NTA analysis showed that the isolated myotube EVs were approximately 100 nm in diameter (Figure S5B), and TEM showed the typical EV morphology (Figure S5C). Western blot analysis confirmed abundant expression of CD9 and CD81, common EV markers in the collected myotube EVs (Figure S5D). In alignment with in vivo observations, strong fluorescent signals of PKH26 dye were detected in the cytoplasm of MC3T3‐E1 cells following incubation with PKH26‐labeled myotube EVs (Figure S5E). Notably, treatment with ATR‐EVs significantly suppressed mRNA (Figure S5F) and protein (Figure S5G,H) expression of osteogenic differentiation‐related genes in MC3T3‐E1 cells, and ALP staining showed reduced ALP activity compared with cells treated with CON‐EVs (Figure S5I). Together, these results demonstrate that ATR‐EVs directly inhibit osteogenic differentiation in vitro.

### 2.4 MiRNAs Mediate the Inhibitory Effect of Aged‐SKM‐EVs on Bone Formation in Aged Mice

**FIGURE 4:**
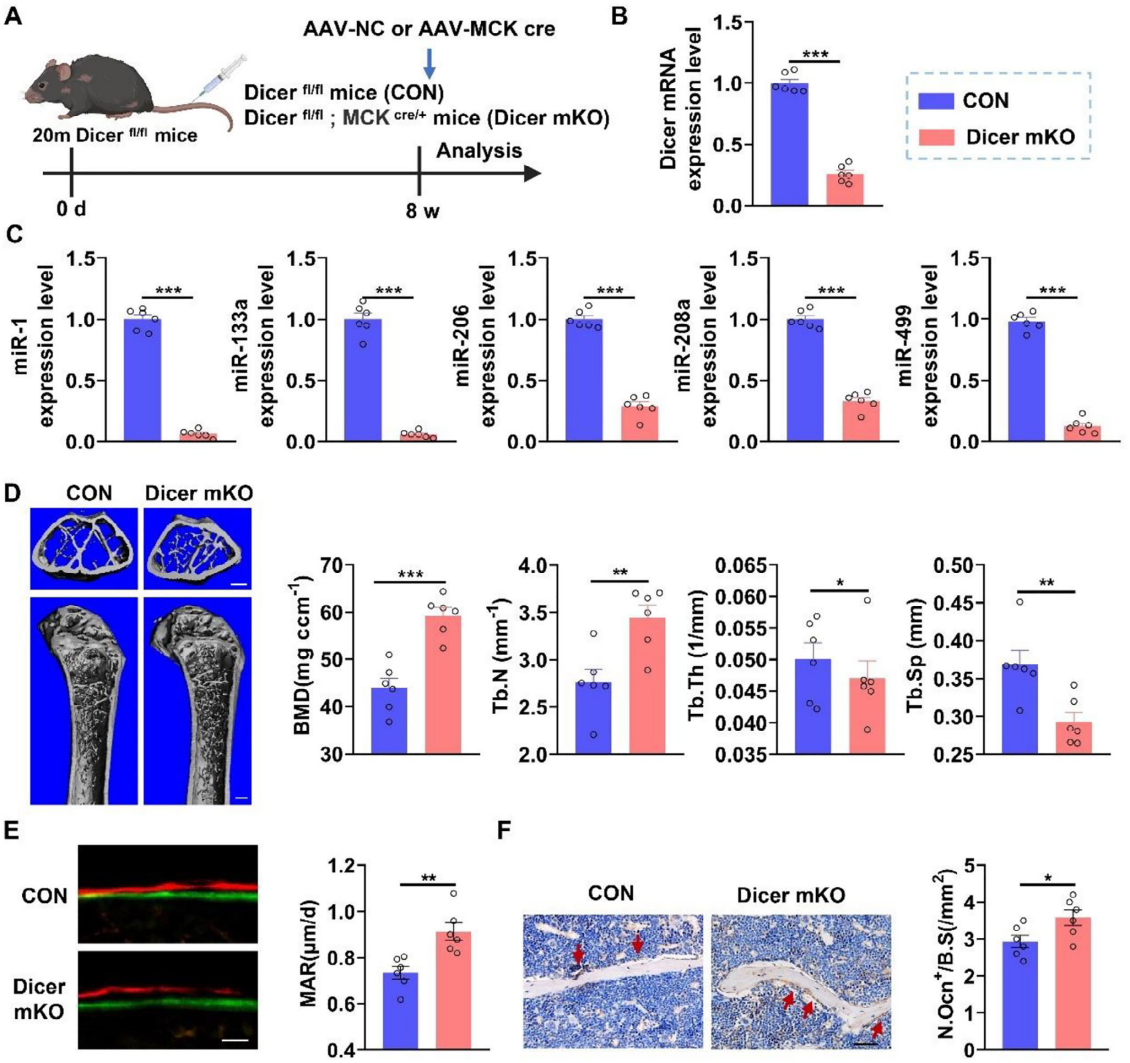
miRNA cargo is responsible for Aged‐SKM‐EVs‐mediated inhibition bone formation in aged mice. (A) Schematic illustration of the experimental design. Aged *Dicer^floxp/floxp^* mice were intravenously injected with AAV‐MCK cre for 8 weeks. (B,C) qPCR analysis of *Dicer* expression and muscle‐specific miRNAs in muscle tissues. *n = 6*. (D) Representative micro‐CT reconstructed images and quantitative analysis of BMD, Tb.N,Tb.Th and Tb.Sp at the distal femoral metaphysis. Scale bar, 100 µm (upper panels) and 500 µm (lower panels). *n = 6*. (E) Representative images of double fluorochrome labeling with calcein green and xylenol orange at the distal femoral metaphysis and quantitative bone histomorphometric analysis of MAR. Scale bar, 25 µm. *n = 6*. (F) Representative immunohistochemical staining of OCN in distal femoral sections and quantification of Ob.S/BS. Scale bar, 50 µm. *n = 6*. All data are presented as mean ± SEM. *P* values were determined by an unpaired two‐tailed Student's *t* test (B‐F). ^*^P< 0.05, ^**^P< 0.01, ^***^P< 0.001.

MiRNAs carried by SKM‐EVs play a pivotal role in the regulation of recipient tissues [11]. To investigate whether miRNAs are responsible for the inhibitory effect of aged‐SKM‐EVs on bone formation, we selectively knocked down *Dicer*, a key enzyme required for miRNA biogenesis, in skeletal muscle of 20‐month‐old mice by injecting a Cre‐dependent muscle creatine kinase (MCK)‐AAV vector into *Dicer* ^floxp/floxp^ mice (Figure 4A). qPCR analyses showed that AAV‐Mck‐cre effectively inhibited *Dicer* expression in skeletal muscle (Figure 4B). Correspondingly, muscle‐specific miRNA levels in muscle tissue were significantly decreased in MCK^cre^; *Dicer* ^floxp/floxp^ mice (*Dicer* mKO) compared with *Dicer* ^floxp/floxp^ mice (CON) (Figure 4C). Micro‐CT analysis revealed that *Dicer* mKO mice exhibited increased BMD and Tb.N, along with decreased Tb.Sp, compared with control mice (Figure 4D). Calcein double labeling demonstrated a higher MAR in *Dicer* mKO mice (Figure 4E). Furthermore, immunohistochemical staining for OCN showed an expanded area of OCN positivity and elevated osteoblast surface per bone surface (Ob.S/BS) at the distal femur in *Dicer* mKO mice relative to controls (Figure 4F). Notably, muscle fiber CSA was similar between *Dicer* mKO and control mice (Figure S6), indicating that muscle structure was not affected by *Dicer* deletion. Collectively, these results indicate that muscle‐specific *Dicer* knockdown attenuates age‐related bone loss, suggesting that miRNAs within Aged‐SKM‐EVs mediate their inhibitory effects on osteogenesis.

### 2.5 MiR‐125a‐5p is Enriched in Plasma EVs from Sarcopenia Patients and Mediates Inhibition of Osteogenic Differentiation

**FIGURE 5:**
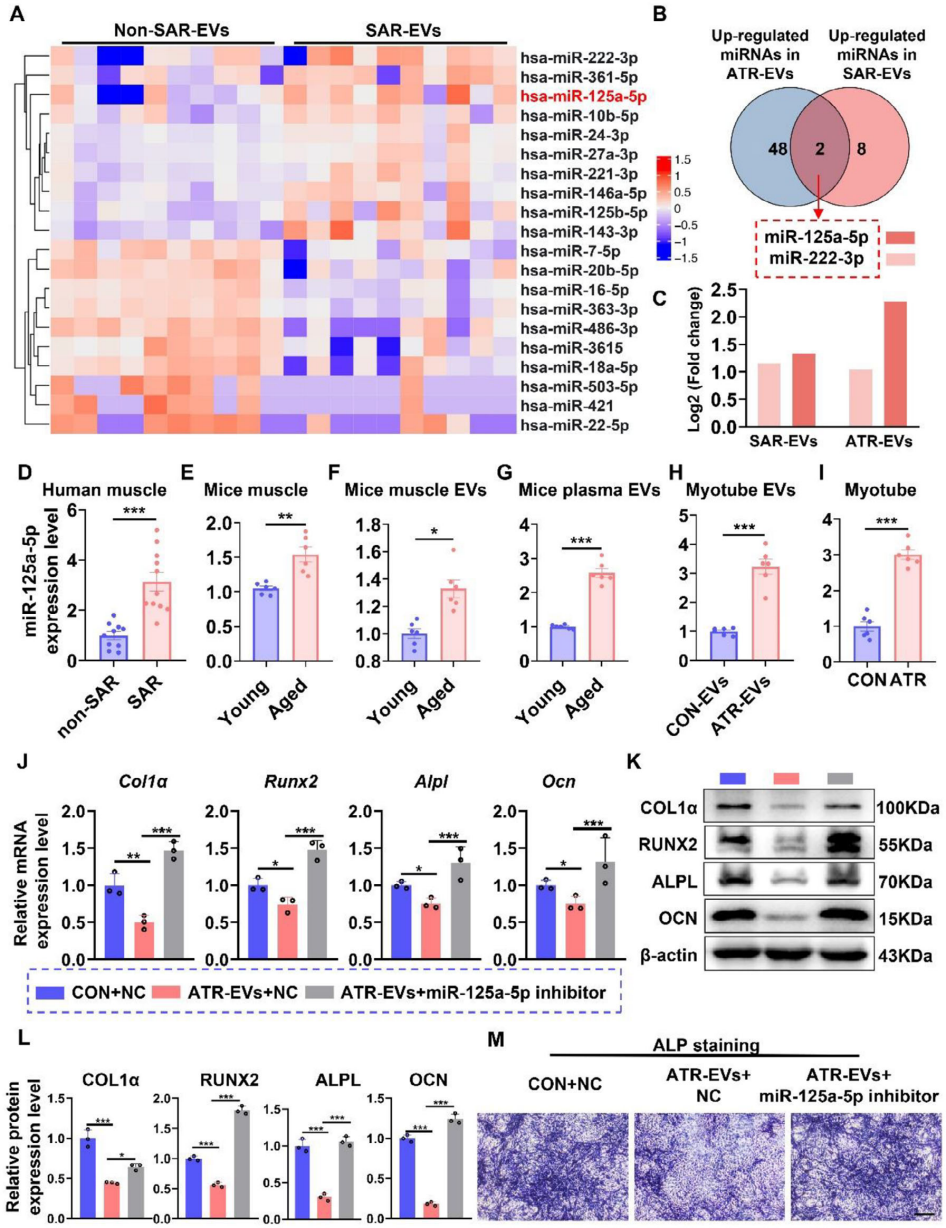
miR‐125a‐5p is enriched in SAR‐EVs and mediates the ATR‐EVs‐induced inhibition of osteogenic differentiation. (A) Heatmap showing differentially expressed miRNAs between SAR‐EVs and non‐SAR‐EVs (top 10 upregulated and downregulated). Biologically independent samples per group. *n = 10*. (B) Venn diagram showing the overlap of upregulated miRNAs in ATR‐EVs and SAR‐EVs. (C) RNA‐seq analysis of miR‐125a‐5p and miR‐222‐3p expression in ATR‐EVs and SAR‐EVs. (D) qPCR analysis of miR‐125a‐5p expression in muscle tissues from sarcopenia patients and non‐sarcopenic subjects (age range: 61 to 79 years). *n = 10*. (E–G) qPCR analysis of miR‐125a‐5p expression in muscle tissues (E), SKM‐EVs (F), and serum EVs (G) from young and aged mice. *n = 6*. (H) qPCR analysis of miR‐125a‐5p expression in CON‐EVs and ATR‐EVs. *n = 3*. (I) qPCR analysis of miR‐125a‐5p expression in CON myotubes and ATR myotubes. *n = 3*. (J) qPCR analysis of osteogenic differentiation‐related genes in preosteoblasts incubated with ATR‐EVs or miR‐125a‐5p inhibitor. *n = 3*. (K,L) Western blot analysis and quantification of osteogenic differentiation‐related protein expression in preosteoblasts incubated with ATR‐EVs or miR‐125a‐5p inhibitor. *n = 3*. (M) ALP staining of osteoblasts after treatment with ATR‐EVs or miR‐125a‐5p inhibitor for 7 days. Scale bars, 500 µm. All data are presented as mean ± SEM. *P* values were determined by unpaired two‐tailed Student's t test (D‐I), or one‐way ANOVA followed by Tukey's multiple comparisons test (J, L). ^*^P< 0.05, ^**^P< 0.01, ^***^P< 0.001.

To investigate the differential expression of microRNAs (miRNAs) in EVs associated with aging‐related muscle atrophy, blood samples were collected from a cohort of 10 sarcopenia patients and 10 age‐matched non‐sarcopenic individuals (61–79 years). Small RNA deep sequencing was performed on serum EVs from non‐sarcopenic individuals (non‐SAR‐EVs) and sarcopenia patients (SAR‐EVs) (Figure S7), as well as CON‐EVs and ATR‐EVs (GSE211885). Analysis identified 10 miRNAs upregulated in SAR‐EVs compared with non‐SAR‐EVs (Figure 5A) and 50 miRNAs upregulated in ATR‐EVs relative to CON‐EVs (Figure S8A). Overlapping analysis revealed miR‐125a‐5p and miR‐222‐3p as common enriched miRNAs in both datasets (Figure 5B), with miR‐125a‐5p exhibiting higher abundance than miR‐222‐3p in SAR‐EVs and ATR‐EVs (Figure 5C). Moreover, miR‐125a‐5p expression in mouse skeletal muscle increased progressively with age (Figure S8B). These findings suggest that miR‐125a‐5p plays an important role in the ATR‐EVs‐mediated inhibition of osteogenic differentiation.

In addition, our findings indicate that miR‐125a‐5p expression in muscle specimens from sarcopenic patients was significantly higher than in non‐sarcopenic individuals, as determined by qPCR analysis (Figure 5D). Furthermore, we corroborated that miR‐125a‐5p expression was significantly higher in the muscle tissues of aged mice relative to young mice (Figure 5E). Similarly, the expression level of miR‐125a‐5p in Aged‐SKM‐EVs was significantly greater than that in EVs from young mice (Figure 5F). Additionally, the expression level of miR‐125a‐5p in serum EVs from aged mice exceeded that in serum EVs from young mice (Figure 5G). Moreover, the expression level of miR‐125a‐5p in EVs released from atrophic myotubes was significantly higher than that in EVs from control myotubes (Figure 5H). Consistently, miR‐125a‐5p expression in atrophic myotubes was also significantly elevated compared to control myotubes (Figure 5I). Collectively, miR‐125a‐5p was identified as one of the most enriched miRNAs in EVs released from aging‐induced atrophic muscle and myotubes.

To further investigate the role of miR‐125a‐5p in mediating the effects of ATR‐EVs, we cultured preosteoblasts with ATR‐EVs in the presence of a miR‐125a‐5p inhibitor. In alignment with prior findings, ATR‐EVs were observed to downregulate the expression of osteogenic differentiation‐related genes at both the mRNA (Figure 5J) and protein levels (Figure S5K,L). Importantly, the introduction of the miR‐125a‐5p inhibitor attenuated the suppressive effects of ATR‐EVs on osteogenic differentiation (Figure 5J–L). Additionally, ALP staining showed that ATR‐EVs significantly decreased ALP activity in preosteoblasts, which was restored by the miR‐125a‐5p inhibitor (Figure 5M). These findings suggest that miR‐125a‐5p functions as a mediator in the ATR‐EVs‐induced suppression of osteogenic differentiation.

### 2.6 Gain‐ and Loss‐of‐Function Experiments Confirm That miR‐125a‐5p in Skeletal Muscle Inhibits Bone Formation during Aging

**FIGURE 6:**
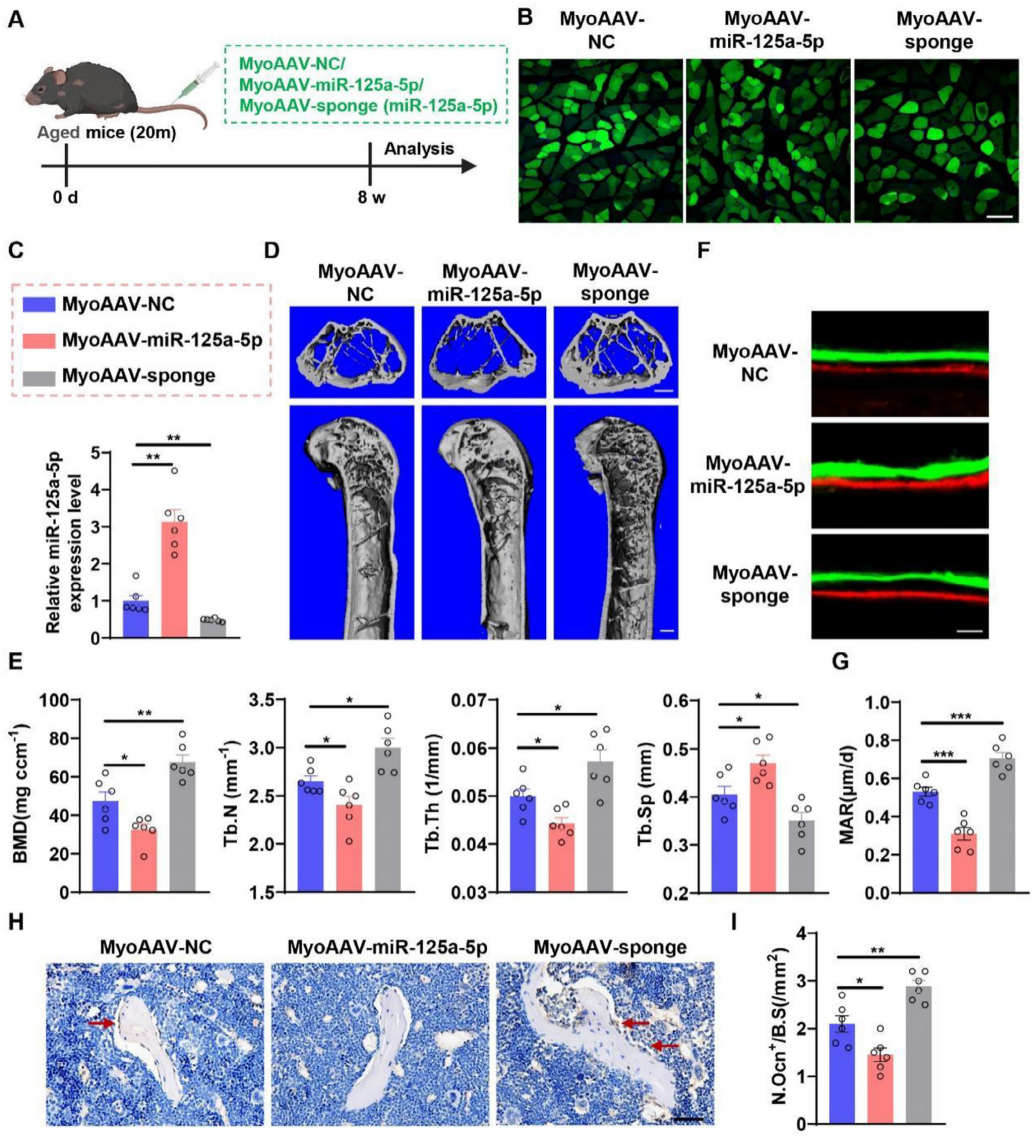
Gain‐ and loss‐of‐function experiments demonstrate that skeletal muscle‐derived miR‐125a‐5p inhibits bone formation during aging. (A) Schematic illustration of the experimental design. Aged mice (20‐month‐old) were intravenously injected with recombinant MyoAAV‐NC, MyoAAV‐miR‐125a‐5p, or MyoAAV‐miR‐125a‐5p‐sponge. (B) Representative fluorescent images of skeletal muscle sections transduced with the indicated MyoAAV vectors. (C) qPCR analysis of miR‐125a‐5p expression in skeletal muscles of mice transduced with the indicated MyoAAV vectors. *n = 6*. (D) Representative micro‐CT reconstructed images of femur. Scale bars, 500 µm. (E) Micro‐CT analysis of BMD, Tb.N, Tb.Th and Tb.Sp in the distal femoral metaphysis. *n = 6*. (F) Representative double labelling with calcein green and xylenol orange in the distal femoral metaphysis. Scale bars, 25 µm. (G) Bone histomorphometric analysis of MAR in the distal femoral metaphysis. *n = 6*. (H) Representative immunohistochemistry staining of OCN in the distal femur. Scale bar, 50 µm. (I) Quantification of Ob.S/BS at the distal femoral metaphysis. *n = 6*. All data are presented as mean ± SEM. *P* values were determined by one‐way ANOVA followed by Tukey's multiple comparisons test (C, E, I). ^*^P< 0.05, ^**^P< 0.01, ^***^P< 0.001.

To further investigate the effect of miR‐125a‐5p‐containing Aged‐SKM‐EVs on bone formation in vivo, we generated recombinant adeno‐associated viruses modified with the RGDLTTP peptide (MyoAAV) for muscle‐specific overexpression or silencing of miR‐125a‐5p in aged mice (Figure 6A) [19]. The MyoAAV vectors co‐expressed GFP, confirming successful muscle transduction (Figure 6B). In mice injected with MyoAAV‐miR‐125a‐5p, skeletal muscle miR‐125a‐5p expression increased 3‐fold, whereas MyoAAV‐miR‐125a‐5p‐sponge reduced miR‐125a‐5p levels by approximately 50% (Figure 6C). Micro‐CT analysis revealed that MyoAAV‐miR‐125a‐5p‐injected mice exhibited lower BMD, Tb.N, and Tb.Th, but higher Tb.Sp in the distal femur compared with controls (Figure 6D,E). Conversely, silencing miR‐125a‐5p via MyoAAV‐miR‐125a‐5p‐sponge improved bone architecture, increasing BMD, Tb.N, Tb.Th, and reducing Tb.Sp (Figure 6D,E). Calcein double labeling showed significantly lower MAR in MyoAAV‐miR‐125a‐5p‐injected mice, whereas silencing miR‐125a‐5p enhanced MAR (Figure 6F,G). Immunohistochemical staining for OCN revealed OCN‐positive area and Ob.S/BS in MyoAAV‐miR‐125a‐5p‐injected mice, whereas MyoAAV‐miR‐125a‐5p‐sponge treatment increased OCN‐positive staining and Ob.S/BS at the distal femur (Figure 6H,I). Analysis of muscle morphology showed that MyoAAV‐miR‐125a‐5p‐injected‐mice exhibited reduced muscle fiber CSA, whereas MyoAAV‐miR‐125a‐5p‐sponge mitigated age‐related muscle atrophy (Figure S9). We further administered MyoAAV‐miR‐125a‐5p injections to 3‐month‐old mice. Similar to the observations in aged mice, the overexpression of miR‐125a‐5p in the skeletal muscle of young mice resulted in significant phenotypic alterations, notably induced muscle atrophy and inhibited bone formation (Figure S10). Collectively, these findings demonstrate that muscle‐specific overexpression of miR‐125a‐5p impairs bone formation, accelerates bone loss, and exacerbates muscle atrophy. Conversely, silencing miR‐125a‐5p promotes bone formation and alleviates both bone loss and muscle atrophy during aging.

### 2.7 MiR‐125a‐5p Inhibits Osteogenic Differentiation by Targeting SIRT7‐Mediated *Sp7* Deacetylation and Suppresses Bone Formation

**FIGURE 7:**
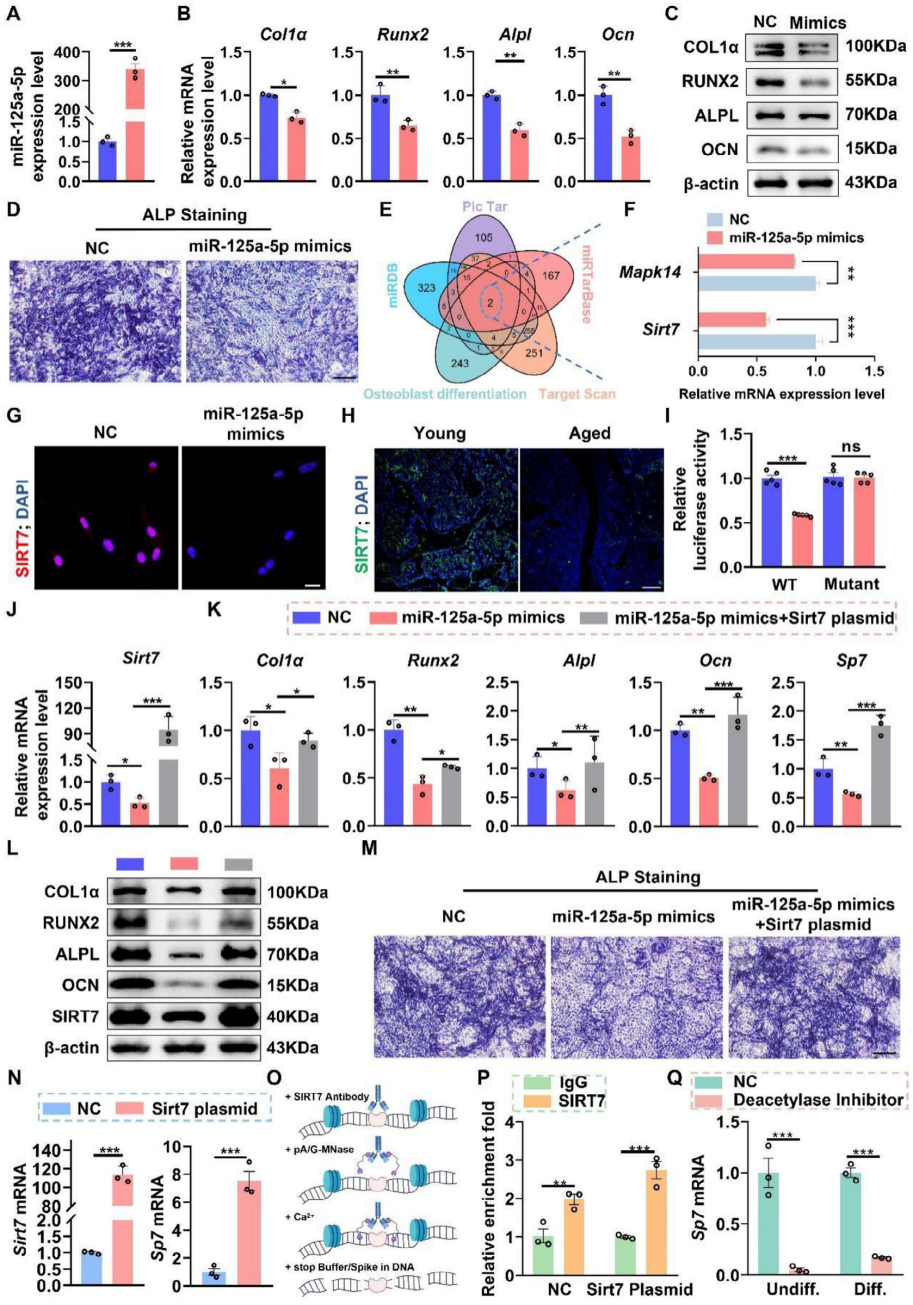
miR‐125a‐5p inhibits osteoblast differentiation by targeting *Sirt7*‐mediated *Sp7* deacetylation. (A) qPCR analysis of miR‐125a‐5p expression in preosteoblasts transfected with miR‐125a‐5p mimics or NC. *n = 3*. (B) qPCR analysis of osteogenic differentiation‐related genes in preosteoblast transfected with miR‐125a‐5p mimics or NC. *n = 3*. (C) Western blot analysis of osteogenic differentiation‐related protein expression in preosteoblasts transfected with miR‐125a‐5p mimics or NC. (D) ALP staining of osteoblasts after transfection with miR‐125a‐5p mimics or NC for 7 days. Scale bars, 500um. (E) Venn diagram showing overlapping predicted targets of miR‐125a‐5p from multiple databases. (F) qPCR analysis of *Sirt7* and *Mapk14* mRNA expression levels in preosteoblasts transfected with miR‐125a‐5p mimics or NC. *n = 3*. (G) Representative immunofluorescence of SIRT7 in preosteoblasts transfected with miR‐125a‐5p mimics or NC. Scale bar, 50 µm. (H) Representative immunofluorescence images of SIRT7 in the distal femur of mice. Scale bar, 100 µm. (I) The dual‐luciferase reporter assay for fluorescent activity of *Sirt7* in HK293T cells after co‐transfection with the WT or mutant SIRT7‐3′‐UTR luciferase construct in combination with miR‐125a‐5p mimics or NC. *n = 5*. (J) qPCR analysis of *Sirt7* mRNA expression in preosteoblasts transfected with NC, miR‐125a‐5p mimics, or miR‐125a‐5p mimics plus Sirt7 plasmid. *n = 3*. (K) qPCR analysis of osteogenic differentiation‐related genes in preosteoblasts transfected with NC, miR‐125a‐5p mimics, or miR‐125a‐5p mimics plus Sirt7 plasmid. *n = 3*. (L) Western blot analysis of SIRT7 and osteogenic differentiation‐related proteins in preosteoblasts transfected with NC, miR‐125a‐5p mimics, or miR‐125a‐5p mimics plus Sirt7 plasmid. (M) ALP staining of preosteoblasts transfected with NC, miR‐125a‐5p mimics, or miR‐125a‐5p mimics plus Sirt7 plasmid for 7 days. Scale bars, 500um. (N) qPCR analysis of *Sirt7* and *Sp7* mRNA expression in preosteoblasts transfected with NC or Sirt7 plasmid. *n = 3*. (O) Schematic representation of CUT&RUN assay using anti‐SIRT7 antibody, followed by DNA purification and qPCR analysis. (P) CUT& RUN‐qPCR analysis showing *Sp7* enrichment by SIRT7 antibody. *n = 3*. (Q) qPCR analysis of *Sp7* mRNA expression in preosteoblasts treated with a deacetylase inhibitor. *n = 3*. All data are presented as mean ± SEM. *P* values were determined by unpaired two‐tailed Student's *t* test (A, B, F, I, N, P, Q), or one‐way ANOVA followed by Tukey's multiple comparisons test (J). ^*^P< 0.05, ^**^P< 0.01, ^***^P< 0.001.

To investigate the functional role of EV miR‐125a‐5p in osteogenic differentiation, preosteoblasts were transfected with either miR‐125a‐5p mimics or a negative control (NC). Transfection with miR‐125a‐5p mimics resulted in a dramatic upregulation of miR‐125a‐5p expression in preosteoblasts (Figure 7A). The overexpression of miR‐125a‐5p significantly suppressed both mRNA (Figure 7B) and protein expression levels (Figure 7C; Figure S11A) of osteogenic differentiation‐related genes. Consistently, ALP staining revealed a significant decrease in ALP activity in preosteoblasts transfected with miR‐125a‐5p mimics (Figure 7D). Collectively, these results indicate that miR‐125a‐5p effectively inhibits osteogenic differentiation.

To identify the target genes of miR‐125a‐5p that contribute to the impairment of osteoblast differentiation, a comprehensive approach was employed. By integrating the osteoblast differentiation‐related gene set (GO:0001649) from the Molecular Signatures Database (MSigDB) with data from multiple databases, including Targetscan, miRDB, miRTarBase, and PicTar, two candidate genes, *Sirt7* and *Mapk14*, were identified (Figure 7E). Subsequent qPCR analysis revealed that *Sirt7* expression was more profoundly suppressed in preosteoblasts transfected with miR‐125a‐5p mimics (Figure 7F). Therefore, *Sirt7* was selected for further investigation. Immunofluorescent analysis revealed a significant reduction in SIRT7 protein levels following miR‐125a‐5p overexpression (Figure 7G). Consistent with these in vitro results, SIRT7 expression was significantly lower in femoral bone tissues from aged mice compared with young mice (Figure 7H).

To determine whether miR‐125a‐5p directly targets *Sirt7*, the predicted miR‐125a‐5p binding site within the 3′‐UTR of *Sirt7* was mutated (Figure S11B). Both wild‐type and mutant Sirt7 sequences were subcloned into the psiCHECK‐2 vector, and luciferase reporter assays were performed in HEK293T cells. The dual‐luciferase reporter assays revealed that miR‐125a‐5p mimics specifically suppressed the luciferase activity of the wild‐type reporters, but not that of the mutant reporter (Figure 7I), indicating that miR‐125a‐5p directly interacts with the binding sequence in the 3′‐UTR of *Sirt7*.

To further assess whether miR‐125a‐5p inhibits osteogenic differentiation through *Sirt7* suppression, preosteoblasts were transfected with miR‐125a‐5p mimics alone or co‐transfected with a *Sirt7* overexpression plasmid. While miR‐125a‐5p overexpression significantly reduced *Sirt7* expression, co‐transfection with the Sirt7 plasmid significantly restored *Sirt7* levels (Figure 7J). Importantly, restoration of *Sirt7* reversed the inhibitory effects of miR‐125a‐5p on osteogenic differentiation, as evidenced by increased mRNA (Figure 7K) and protein expression (Figure 7L; Figure S11C) of osteogenic markers. ALP staining further confirmed a significant recovery of ALP activity in cells co‐transfected with miR‐125a‐5p mimics and the *Sirt7* plasmid compared with cells transfected with miR‐125a‐5p mimics alone (Figure 7M). Conversely, *Sirt7* knockdown using siRNA significantly reduced the expression of osteogenic genes, including *Sp7*, and significantly decreased ALP activity, indicating impaired early osteogenic commitment (Figure S11D–H).

Mechanistically, *Sirt7* overexpression significantly increased *Sp7* mRNA expression (Figure 7N). CUT&RUN analysis further demonstrated direct binding of SIRT7 to the *Sp7* promoter, which was significantly enhanced upon *Sirt7* overexpression (Figure 7O, P). Moreover, pharmacological inhibition of deacetylase activity in MC3T3‐E1 cells resulted in a pronounced reduction in *Sp7* transcription under both undifferentiated and osteogenic conditions (Figure 7Q), accompanied by downregulation of *Col1a1* and *Runx2* (Figure S11I). These findings indicate that SIRT7‐dependent histone deacetylation is essential for maintaining *Sp7* transcription and early osteogenic differentiation. In summary, these results demonstrate that miR‐125a‐5p inhibits osteogenic differentiation in preosteoblasts by directly targeting *Sirt7*. This disruption of SIRT7‐mediated histone deacetylation at the *Sp7* promoter suppresses *Sp7* transcription, ultimately leading to impaired bone formation during aging.

## 3 Discussion

During aging, skeletal muscle atrophy and senile osteoporosis frequently occur in parallel, profoundly compromising quality of life and significantly increasing fracture risk in the elderly [21]. The maintenance of normal skeletal muscle mass and function is essential for preserving typical bone morphology and function. Muscle atrophy or impairment, whether due to aging, trauma, or inflammation, can lead to the onset of osteoporosis [22]. However, the precise regulatory mechanisms by which atrophic skeletal muscle affects bone formation during aging remain poorly elucidated. In the present study, we demonstrate that during aging, aged‐SKM‐EVs enter the circulation, accumulate in bone tissue, and are efficiently internalized by osteoblast‐lineage cells, thereby inhibiting bone formation. Mechanistically, these aged‐SKM‐EVs suppress osteogenic differentiation of osteoprogenitor cells through delivery of miR‐125a‐5p, revealing a previously unrecognized endocrine mechanism by which aged muscle actively impairs bone anabolism.

Accumulating evidence indicates that skeletal muscle [8, 23], myoblasts [10, 24], and myotubes are capable of secreting EVs [25], which mediate the transfer of proteins, mRNA, and miRNA to recipient cells and thereby regulate diverse biological functions. Numerous studies have further demonstrated that EVs exert biological effects in distant tissues through the circulatory system [26, 27]. For instance, Hala Aswad et al. reported that fluorescently labeled muscle‐derived EVs, when intravenously injected into C57BL/6 mice, distributed to multiple organs within 24 h, including the lungs, liver, spleen, brain, heart, pancreas, and gastrointestinal tract, although bone tissue was not examined [28]. In addition, Shixing Ma et al. demonstrated that SKM‐EVs can infiltrate bone tissue and be internalized by bone marrow stromal cells, thereby modulating bone metabolism [29]. Nevertheless, the role of SKM‐EVs in bone metabolism during aging remains poorly understood.

In this study, we successfully developed skeletal muscle‐specific EV tracer transgenic mice for the first time. Utilizing this model, we found that aging significantly increases skeletal muscle EV release, which can circulate and localize to bone tissue, particularly within osteoblasts. Characterization of the isolated EVs based on morphology, size, and marker protein expression revealed a substantial enrichment of EVs. To selectively inhibit EV generation in skeletal muscle, we designed a tail vein‐delivered, muscle‐targeted liposomal system encapsulating the neutral sphingomyelinase (nSMase) inhibitor GW4869. It is important to note that GW4869 inhibits reversible nSMase, and intraperitoneal administration in vivo has been reported to produce nonspecific effects, including variable impacts on body weight [30, 31, 32]. By using a muscle‐targeted delivery system, we minimized these off‐target effects and specifically suppressed EV production in skeletal muscle. Our findings indicate that this approach effectively inhibits the generation of EVs in the muscles of aged mice and mitigates age‐related bone loss. Additionally, the administration of Aged‐SKM‐EVs into young mice significantly impeded bone formation, leading to a reduction in bone mass. Collectively, these findings demonstrate that Aged‐SKM‐EVs can enter bone tissue, be internalized by osteoblasts, and inhibit osteoblast differentiation, thereby accelerating bone loss during aging.

EVs carry diverse bioactive cargos, including miRNAs, other types of RNA, lipids, and proteins [33]. Among these cargos, miRNAs are small noncoding RNAs of approximately 19–25 nucleotides and are widely recognized as key functional mediators of EV‐induced biological effects [32]. EV‐encapsulated miRNAs can be released into the circulation and participate in inter‐organ communication by regulating gene expression in distant target cells [34]. To specifically evaluate the contribution of muscle‐derived miRNAs, we generated aged mice with skeletal muscle‐specific deletion of *Dice*r, a critical enzyme for miRNA biogenesis [35]. Importantly, muscle‐specific *Dicer* ablation significantly attenuated bone loss in aged mice, indicating that miRNAs packaged within Aged‐SKM‐EVs play a crucial role in age‐related bone loss. RNA sequencing analysis of plasma EVs from elderly individuals with or without sarcopenia revealed that miR‐125a‐5p was significantly enriched in EVs from sarcopenic patients. Consistently, miR‐125a‐5p expression was significantly elevated in muscle biopsy specimens, muscle‐derived EVs, and circulating EVs from aged mice, as well as in atrophic myotubes and their derived EVs in vitro. These findings identify miR‐125a‐5p as a key EV‐associated miRNA released from atrophic skeletal muscle into the circulation. Importantly, inhibition of miR‐125a‐5p effectively reversed the suppressive effects of Aged‐SKM‐EVs on osteogenic differentiation, indicating that miR‐125a‐5p is a major functional mediator of this process.

Previous studies have shown that miR‐125a‐5p is induced under oxidative stress conditions and suppresses osteogenic differentiation by inhibiting VEGF expression in human adipose‐derived mesenchymal stem cells [36]. However, its role in osteoblast‐mediated bone formation in vivo has remained unclear. In our study, we employed a skeletal muscle‐targeted recombinant AAV system to manipulate miR‐125a‐5p expression in vivo. Muscle‐specific overexpression of miR‐125a‐5p significantly impaired bone formation and accelerated bone loss in young and aged mice, whereas silencing miR‐125a‐5p in atrophic skeletal muscle promoted osteogenesis and alleviated age‐related bone loss. Notably, miR‐125a‐5p overexpression also exacerbated muscle atrophy, while its inhibition attenuated muscle wasting, indicating that skeletal muscle‐derived miR‐125a‐5p functions as a dual regulator of musculoskeletal aging.

Mechanistically, we identified Sirtuin 7 (SIRT7) as a direct and functionally relevant target of miR‐125a‐5p. SIRT7 is a NAD^+^‐dependent histone deacetylase that regulates diverse cellular processes, including aging [37], metabolism, genomic stability, and differentiation [38, 39]. Previous research has established a critical role for SIRT7 in osteoblast biology [40]. Notably, SIRT7 regulates bone formation by modulating the lysine acetylation of SP7/Osterix in osteoblasts, and osteoblast‐specific *Sirt7* knockout mice exhibit severe bone loss, indicating its critical role in osteoblast‐mediated bone formation [41]. Our study demonstrates that miR‐125a‐5p directly suppresses *Sirt7* expression in osteoprogenitor cells, thereby impairing SIRT7‐mediated histone deacetylation at the *Sp7* promoter and attenuating *Sp7* transcription. Importantly, restoration of SIRT7 expression rescues *Sp7* activity and reverses the inhibitory effects of miR‐125a‐5p on osteogenic gene expression, indicating that miR‐125a‐5p suppresses osteogenesis predominantly through disruption of the SIRT7‐*Sp7* transcriptional regulatory axis.

Additional limitations of the present study remain unresolved. First, aged skeletal muscle secretes not only miR‐125a‐5p but also a variety of senescence‐associated factors [42], including senescence‐associated secretory phenotype (SASP) components [43], pro‐inflammatory cytokines, and damage‐associated molecular patterns (DAMPs). These factors may be co‐packaged within EVs, thereby impairing bone formation and contributing to systemic aging, including skeletal aging. While our study identifies miR‐125a‐5p as a key mediator linking atrophic muscle to impaired osteogenesis, it likely acts in concert with other factors in Aged‐SKM‐EVs. Second, the clinical cohort in this study consisted exclusively of male participants, which may limit the generalizability of the findings. Future studies incorporating female cohorts will be required to address potential sex‐specific differences in EV‐mediated muscle‐bone crosstalk during aging. Third, GW4869 inhibits multiple EV subpopulations, and our results therefore reflect the combined effects of these EVs. Future studies should aim to distinguish the contributions of specific EV subtypes using density gradient ultracentrifugation or immunoaffinity‐based isolation strategies. In addition, comprehensive characterization of EV cargos, including proteins, cytokines, and miRNAs, will be essential for elucidating their precise effects on bone metabolism and inter‐organ aging.

In summary, our study identifies a previously unrecognized muscle‐bone endocrine axis during aging, in which miR‐125a‐5p carried by Aged‐SKM‐EVs inhibits osteogenic differentiation by suppressing SIRT7‐mediated histone deacetylation at the *Sp7* promoter, thereby attenuating Sp7 transcription. These findings establish Aged‐SKM‐EVs and their miRNA cargo as critical mediators of musculoskeletal crosstalk in senile osteoporosis and suggest that targeting Aged‐SKM EVs or miR‐125a‐5p may represent a promising therapeutic strategy for the simultaneous treatment of sarcopenia and osteoporosis.

## 4 Experimental Section

### 4.1 Animal Models and Treatments

All animals were housed in a specific pathogen‐free (SPF) facility in individually ventilated cages under controlled environmental conditions, including a temperature of 20–24°C, relative humidity of 30–70%, and positive air pressure. Animals were maintained on a 12 h light/dark cycle with free access to food and water. All experimental procedures were approved by the Institutional Animal Care and Use Committee (IACUC) and conducted in accordance with the Animal Care and Use Guidelines of Nanjing Drum Tower Hospital, Nanjing University Medical School (approval no. 20201220). Male C57BL/6J mice (2 months old) were obtained from GemPharmatech Co., Ltd and aged to 3–24 months for experimental use.

Skeletal muscle‐specific Cd63 reporter mice were generated by crossing *Cd63^(loxp‐eGFP)^* mice with *HSA^Cre^* mice to obtain *HSA^Cre^*; *Cd63* ^(^ *^loxp^* ^−^ *^eGFP^* ^)^ mice. Conditional skeletal muscle‐specific *Dicer* knockout mice (hereafter referred to as Dicer mKO) were generated by systemic delivery of MCK‐Cre AAV (6.0E+11VG) via tail vein injection into 20‐month‐old *Dicer^fl/fl^* mice. Control mice received an equivalent dose of negative control AAV. All mouse lines were maintained on a C57BL/6 background. 6‐ and 24‐month‐old *HSA^Cre^*; *Cd63* ^(^ *^loxp^* ^−^ *^eGFP^* ^)^ mice, 22‐month‐old *Dicer* mKO mice, and wild‐type littermate controls were used for experiments. Genotyping was performed by PCR, with primer sequences listed in Table S1. *Cd63^(loxp‐eGFP)^* mice were obtained from Shanghai Model Organisms, and *Dicer^fl/fl^* mice were purchased from GemPharmatech. *HSA^cre^* mice were kindly provided by Professor Zhenji Gan.

To specifically block EV generation in skeletal muscle, 24‐month‐old C57BL/6J male mice were administered the neutral sphingomyelinase 2 inhibitor GW4869 (2.5 mg/kg body weight, 3 times per week, HY‐19363, MedChemExpress) using a skeletal muscle‐targeted liposome delivery system (R‐LS‐GW4869) via tail vein injection for 6 weeks. To assess the effects of Aged‐SKM‐EVs on bone formation, EVs isolated from the skeletal muscle tissue of 24‐month‐old mice were sterilized through a 0.22 µm filter and intravenously injected into 3‐month‐old male C57BL/6J mice (100 µg per mouse, three times per week) for 4 weeks.

For skeletal muscle‐specific overexpression or silencing of miR‐125a‐5p, a muscle‐targeting peptide motif (RGDLTTP) was incorporated into recombinant AAV9 vectors (MyoAAV). Myo‐miR‐125a‐5p, Myo‐miR‐125a‐5p‐sponge, and Myo‐negative control (NC) constructs, each expressing GFP, were generated by OBiO Technology (Shanghai, China). Aged mice (20 months) received intravenous injections of 6.0E+11 VG [44], while young mice (3 months) received 4.0E+11 VG [19].

### 4.2 Human Specimens

The study involving human specimens was approved by the Medical Ethics Committee of the Affiliated Drum Tower Hospital, Nanjing University Medical School, in accordance with the Declaration of Helsinki (2020‐323‐01). All participants provided written informed consent. The subjects analyzed were older men without sarcopenia (70 ± 2.11 years, *n* = 10) and those with sarcopenia (72 ± 4.78 years, *n* = 10). Participants were excluded if they had any metabolic diseases, liver or renal diseases, or inflammatory disorders. Sarcopenia was diagnosed according to the criteria of the Asian Working Group for Sarcopenia (AWGS), defined by the presence of both low muscle mass and low muscle strength, assessed by calf circumference (<34 cm) and handgrip strength (<28 kg) in men. Muscle specimens were collected from the gluteus medius of participants during elective orthopedic surgery. Whole blood samples were collected in EDTA plasma tubes and processed for plasma isolation within 2 h, after which plasma was stored at −80°C. Detailed information on all subjects is provided in Table S2.

### 4.3 Cell Culture and Treatment

Mouse C2C12 myoblasts (CRL‐1772, ATCC, RRID: CVCL_0188) and human HEK293T cells (CRL‐3216, ATCC, *RRID*: CVCL_0063) were cultured in DMEM (319‐027‐CL, WISENT) supplemented with 10% fetal bovine serum (FBS, BC‐SE‐FBS07, Bio‐Channel Biological Technology Co., Ltd) and 1% penicillin‐streptomycin, and maintained at 37°C in a humidified incubator with 5% CO_2_. Cell cultures were either passaged using a trypsin‐EDTA solution (0.25%) or cryopreserved in a serum‐free cell freezing medium (BMU108, SuperKine) using cryovials (606802, NEST Biotechnology).

For myogenic differentiation, myoblasts were induced by switching to differentiation medium (DMEM containing 2% horse serum; HyClone, Cytiva) when they reached 80–90% confluence. The culture medium was changed every 2 days, and differentiation was allowed to proceed for 5 days. Myotube atrophy was induced by starvation, achieved by incubating the myotubes in Earle's Balanced Salt Solution (EBSS, H2025, Solarbio) for 7 h at 37°C. The myotubes were then fixed in 4% paraformaldehyde (PFA) for 15 min and stained with DAPI for nuclear visualization. Green phalloidin was used to stain the osteoblast cytoskeleton.

Primary osteoblastic cells were isolated from the calvaria of neonatal mice (48–72 h after birth). The calvaria were dissected, rinsed with PBS, and digested in trypsin‐EDTA (H0516, HAKATA) at 37°C for 10 min. After the initial digestion, the tissue was rinsed with α‐MEM and digested twice more in freshly prepared 0.1% collagenase A (4155743, Roche, Basel, Switzerland) in α‐MEM. After digestion, the supernatants were collected, combined, and centrifuged to pellet the cells. The primary osteoblastic cells were cultured in α‐MEM supplemented with 50 µg/mL ascorbic acid and 10% FBS for 40 min. For routine culture, primary osteoblastic cells and the mouse preosteoblast cell line MC3T3‐E1 (IM‐M080, IMMOCELL, *RRID*: CVCL_5437) were maintained in α‐MEM supplemented with 10% FBS and 1% penicillin‐streptomycin at 37°C in a 5% CO2 incubator.

For osteogenic differentiation, both primary osteoblastic cells and MC3T3‐E1 cells were cultured in osteogenic differentiation medium (EOMX‐D101, Haixing Biosciences) containing 10% FBS, 10 nmol/L dexamethasone, 10 mmol/L β‐glycerophosphate disodium salt pentahydrate, and 50 µg/mL vitamin C. The osteogenic induction was maintained for 14 days, with the differentiation medium replaced every 2 days. ALP activity was assessed on day 14 using the BCIP/NBT Alkaline Phosphatase Color Development Kit (C3206, Beyotime). Briefly, cells were fixed and incubated with BCIP/NBT working solution at room temperature in the dark until sufficient color development was observed.

For the co‐culture experiments, myotubes were cultured in the upper chambers of 12‐well Transwell inserts (1101100, SAINING Biotechnology). Myotubes were divided into a control group, an atrophy group, and an atrophy group pretreated with GW4869 20 µм for 24 h. After pretreatment, the culture medium was replaced, and the myotubes were co‐cultured with MC3T3‐E1 cells seeded in the lower chambers.

For miRNA, plasmid, and siRNA transfections, MC3T3‐E1 cells were seeded in 12‐well plates and cultured until they reached 70–80% confluency. Cells were then transfected with miRNA‐125a‐5p mimics (Ribo, Guangzhou, China) at 50 nм, miRNA‐125a‐5p inhibitors (Ribo, Guangzhou, China) at 100 nм, *si‐Sirt7* at 50 nм (Generay Biotech, Shanghai, China), Sirt7 plasmid (1.25 µg/mL), or negative control using Lipofectamine 2000 (Invitrogen, Thermo Fisher Scientific). The culture medium was replaced with fresh medium 8 h after transfection. HEK293T cells were used for luciferase reporter assays. The *si‐Sirt7* primer sequences (Generay Biotech, Shanghai, China) are provided in Table S3.

To investigate the effect of deacetylation on *Sp7*, MC3T3‐E1 cells were seeded in 12‐well plates and cultured until they reached 70–80% confluency. A deacetylase inhibitor (P1112, Beyotime) was then added, and cells were incubated for 48 h in either growth medium (Undiff.) or osteogenic differentiation medium (Diff.).

All cell lines were authenticated by morphology and growth characteristic examination, and were routinely tested to confirm the absence of mycoplasma contamination prior to experiments.

### 4.4 EVs Isolation, Characterization, and Trafficking Assays

For tissue‐derived EV isolation, fresh skeletal muscle tissues from young and aged mice were rinsed with ice‐cold PBS and cut into 2 × 2 × 2 mm^3^ fragments using a sterile scalpel to remove connective tissue and blood contamination. The tissue fragments were then placed in DMEM medium (1 mL/0.2 g tissue). The fragments were transferred to culture plates and digested with collagenase D (final concentration: 2 mg/mL) and DNase I (final concentration: 40 U/mL) in 2 mL DMEM per well at 37°C under gentle agitation for 30 min. The resulting tissue suspension was filtered through a sterile 70‐µm cell strainer into a 50‐mL tube. Wells were washed with PBS (1 mL/well) and passed through the strainer to recover any remaining EVs. For myotube‐derived EV collection, myotube cells were cultured in DMEM containing 10% EV‐depleted FBS (FBS‐EX50, NEWZERUM Ltd) or EBSS at 37°C with 5% CO_2_. Tissue digests, culture supernatants, or serum samples were centrifuged sequentially at 300 × g for 10 minutes and 2,000 × g for 20 minutes at 4°C to remove dead cells and debris. The supernatant was then centrifuged at 10,000 × g for 30 minutes to remove large particles and organelles. The resulting supernatant was filtered through a 0.22‐µm membrane and ultracentrifuged at 100,000 × g for 70 minutes at 4°C (Beckman‐Coulter Optima L‐80‐XP ultracentrifuge, Type 70 Ti rotor). The EV pellet was resuspended in PBS and further ultracentrifuged at 100,000 × g for an additional 70 minutes. The final EV pellet was resuspended in PBS for subsequent experiments.

For EV characterization, the protein content of EVs was quantified using a BCA protein assay kit (Beyotime, Nanjing, China). Western blotting was performed to detect EV‐specific markers, including CD81, CD9, CD63, and TSG101. The size distribution and concentration of EVs were determined using NTA. EV morphology was examined using TEM.

For in vitro EV uptake assays, EVs were labeled with PKH26 red fluorescent dye (P0096, Merck) according to the manufacturer's instructions and added to osteoblast cells, followed by incubation at 37°C for 24 h. Additionally, myotube‐derived EVs were fluorescently labeled by transfecting CMV‐GFP‐CD63 into myotubes. CMV‐GFP‐CD63‐transfected myotubes were co‐cultured with osteoblast cells at 37°C for 3 days. After incubation, the culture supernatant was discarded, and cells were washed with PBS. The cells were fixed in 4% PFA for 15 minutes and stained with DAPI for nuclear visualization. Green phalloidin was used to stain the osteoblast cytoskeleton. For in vivo EV uptake assays, PKH26‐labeled EVs (100 µg per mouse) were injected into mice via the tail vein. After 24 h, femurs were collected for further analysis.

### 4.5 RNA Extraction, Reverse Transcription, and Real‐Time Quantitative PCR

Total RNA was extracted from tissues and cultured cells using TRIzol reagent (CW0580S, CWBIO) according to the manufacturer's protocol. EV‐derived miRNAs were isolated using the miRNeasy Kit (QIAGEN). For mRNA reverse transcription, first‐strand cDNA was synthesized from total RNA using the HiScript III RT SuperMix (R323‐01, Vazyme Biotech Co.,Ltd). Real‐time PCR was performed on an ABI ViiA 7 system (Thermo, USA) with ChamQ SYBR qPCR Master Mix (Q711, Vazyme Biotech Co.,Ltd). The mRNA primer sequences (Generay Biotech, Shanghai, China) are provided in Table S4. Mature miRNAs expression was quantified using the Hairpin‐it MicroRNA qPCR Quantification Kit with U6 snRNA normalization (RiboBio, China), with miRNA primers sourced from RiboBio.

### 4.6 Western Blot and Antibodies

Total protein was extracted from tissue samples, cell samples, and EVs using the Protein Extraction Kit (KGB5303‐100, KeyGEN BioTech). Protein concentrations were determined using the BCA Protein Assay Kit (Beyotime, Nanjing, China). Proteins were denatured by boiling at 95°C for 5 min in 5× SDS‐PAGE loading buffer. (BOSTER, China). The denatured proteins were subjected to electrophoresis on 10% or 12.5% SDS‐PAGE gels and then transferred to polyvinylidene difluoride (PVDF) membranes (Bio‐Rad, CA). The membranes were incubated with primary antibodies, as listed in Table S5. Following this, the membranes were treated with horseradish peroxidase (HRP)‐conjugated secondary antibodies (Proteintech, China). Signals were visualized using an Enhanced Chemiluminescence (ECL) substrate (Tanon, China). The primary antibodies used were as follows: RAB27A (A23993, ABclonal), CD9 (20597‐1‐AP, Proteintech), CD81 (66866‐1‐lg, Proteintech), CD63 (ab252919, Abcam), TSG101 (ab125011, Abcam), OCN (A6205, ABclonal), COL1α1 (A1352, ABclonal), RUNX2 (R25634, Zen‐bio), ALPL (A0514, ABclonal), SIRT7 (12994‐1‐AP, Proteintech), Calnexin (10427020AP, Proteintech), β‐actin (AC026, ABclonal), α‐tubulin (AC012, ABclonal).

### 4.7 Immunofluorescence and Immunohistochemical Staining

Bone tissues were dissected and fixed in 4% paraformaldehyde for 24 h, whereas muscle tissues were fixed for 12 h. Following fixation, femurs were decalcified in 10% EDTA (HY‐Y0682, MedChemExpress) for 28 days and subsequently embedded in paraffin or optimal cutting temperature (OCT) compound (No. 4583, Sakura). Tissue sections (5 µm thick) were prepared for histological and immunostaining analyses. Sections were incubated with primary antibodies overnight at 4°C, followed by incubation with fluorophore‐ or horseradish peroxidase‐conjugated secondary antibodies at room temperature for 1 h in the dark. Muscle sections were additionally subjected to hematoxylin and eosin (H&E) and Masson's trichrome staining to evaluate myofiber cross‐sectional area (CSA) and fibrosis. Quantification of CSA and fibrotic area was performed using ImageJ software.

For immunofluorescence staining of cells, MC3T3‐E1 cells were cultured on glass‐bottom dishes (801002, NEST Biotechnology) and subjected to the indicated treatments. Cells were fixed with 4% paraformaldehyde for 10 min, permeabilized with 0.1% Triton X‐100 (E‐IR‐R122, Elabscience Biotechnology Co., Ltd), and blocked with goat serum (E‐IR‐R110, Elabscience Biotechnology Co., Ltd) at room temperature for 60 min. Cells were then incubated with primary antibodies overnight at 4°C, followed by incubation with Alexa Fluor 594‐conjugated secondary antibodies (A‐11094 and 331594, Invitrogen). Nuclei were counterstained with DAPI. The primary antibodies used included RAB27A (A23993, ABclonal), CD63 (ab252919, Abcam), OCN (bs‐0470R, Bioss USA), SIRT7 (12994‐1‐AP, Proteintech), and nSMase2 (C01004, Signalway Antibody).

### 4.8 Physiological Muscle Force Generation Assay

Muscle force generation capacity was assessed in the soleus (SOL) and extensor digitorum longus (EDL) muscles using an ASI muscle contraction system (Aurora Scientific). Throughout the procedure, mice were anesthetized with isoflurane. The tibialis anterior (TA) or gastrocnemius (GAS) muscles were excised, followed by dissection of the EDL or SOL muscles. The muscles were adjusted to their optimal length (OL) and tested at various frequencies to determine absolute force values. Muscle force data were recorded and analyzed using Dynamic Muscle Control and GraphPad Prism software.

### 4.9 Micro‐CT Analysis

The left femurs of the mice were dissected and fixed in 4% paraformaldehyde for 24 h. After fixation, the femurs were scanned and analyzed using a micro‐CT system (vivaCT80, SCANCO Medical AG, Switzerland). The region of interest (ROI) was defined as the distal portion of the femur, extending from 0.1 mm below the growth plate to the proximal femur. Quantitative assessments included bone mineral density (BMD), trabecular bone volume relative to tissue volume (Tb.BV/TV), trabecular number (Tb.N), trabecular thickness (Tb.Th), and trabecular separation (Tb.Sp), as determined by Scanco Medical software.

### 4.10 Synthesis and Characterization of Skeletal Muscle‐Targeted Delivery System

RGDLTTP was an effective peptide for targeting skeletal muscle. To create a muscle‐specific delivery system, liposomes were designed to carry GW4869, an inhibitor of extracellular vesicle production. The synthesis process involved dissolving DSPE‐PEG2000 in chloroform and RGDLTTP in methanol, mixing with ammonia, incubating, and using rotary evaporation to form the crystals. The resulting crystals were dissolved in acetonitrile and mixed with a lipid blend of DOTAP, DOPE, cholesterol, and DSPE‐PEG2000 (3:1:1:1). After crystal formation, the mixture was hydrated in PBS, sonicated, and filtered. GW4869 was added, mixed, and filtered again to produce R‐LS‐GW4869 liposomes, which were stored at 4°C. Blank liposomes contained no active ingredients, while R‐LS‐Dir contained DiR at 1 mg/mL. Liposomes were characterized by measuring particle size using a Brookhaven 90 Plus Laser Particle Sizer and analyzing morphology with transmission electron microscopy (TEM). For in vivo targeting efficiency, DiR‐labeled RGDLTTP‐modified or control liposomes were injected into mice via the tail vein. After 48 h, the mice were euthanized, and major organs were collected for fluorescence analysis using an in vivo imaging system.

### 4.11 Small RNA Deep Sequencing and Bioinformatics Analysis

MiRNA components in non‐SAR‐EVs and SAR‐EVs were profiled by single‐end sequencing (1 × 50 bp) on an Illumina HiSeq 2500 at LC‐BIO (Hangzhou, China). The raw data were processed using an in‐house program, ACGT101‐miR (LC Sciences, Houston, Texas, USA), to remove adapter dimers, junk sequences, low‐complexity regions, common RNA families (rRNA, tRNA, snRNA, snoRNA), repeats, and sequences < 18 nt or > 26 nt in length. Subsequently, the unique sequences with lengths ranging from 18–26 nt were mapped to miRNA sequences in miRBase 22.0. Mapping was also performed against the human genome to identify precursor miRNAs (pre‐miRNAs). Unique sequences aligning to known miRNAs in miRBase 22.0 were classified as known miRNAs. The secondary structure of pre‐miRNAs was analyzed, displaying a hairpin conformation, including both 5p‐ and 3p‐derived miRNAs. Unique sequences mapping to the opposite arm of a pre‐miRNA but not annotated in miRBase 22.0 were considered as 5p‐ or 3p‐derived miRNA candidates. The remaining unmapped sequences were further aligned to the human genome to identify potential novel miRNAs. Differential expression analysis of two conditions/groups was performed using the DESeq R package (version 3.0.3). P‐values were adjusted using the Benjamini‐Hochberg method, and a corrected P‐value of 0.05 was set as the threshold for significantly differential expression by default.

### 4.12 Luciferase Reporter Assay

Luciferase reporter plasmids (psiCHECK2) incorporating either the wild‐type (WT) or mutant 3' untranslated region (UTR) of SIRT7 were obtained from Hanheng Biotechnology (Shanghai, China). HEK293T cells were cultured in 96‐well plates, and co‐transfection was performed using Lipofectamine 2000. The cells were transfected with reporter constructs containing either the WT or mutant 3' UTR, with or without the addition of mmu‐miR‐125a‐5p. Following transfection, luciferase activity in the cell lysates was quantified using the Dual‐Glo luciferase assay system (Promega, Madison, WI, USA).

### 4.13 Cleavage Under Targets and Release Using Nuclease (CUT&RUN) Assay

CUT&RUN assays were performed using the Hyperactive pG‐MNase CUT&RUN Assay Kit for PCR/qPCR (HD103, Vazyme Biotech co.,Ltd) according to the manufacturer's instructions. Approximately 5 × 10^5^ cells were used per reaction. Cells were incubated overnight at 4°C with either an IgG control (HA722127, Hangzhou Huaan Biotechnology) or SIRT7 antibody (HA723075, Hangzhou Huaan Biotechnology), followed by the cleavage and release steps. CUT&RUN DNA fragments were purified using the provided DNA purification buffers and spin columns. Finally, DNA products were quantified by qPCR. The *Sp7* primer details are provided in Table S4.

### 4.14 Statistical Analysis

Data were presented as means ± standard error of the mean (SEM). Statistical analyses were conducted using unpaired Student's *t*‐tests or one‐way analysis of variance (ANOVA). All analyses were performed with GraphPad Prism software (version 8.0.1). A *p*‐value of less than 0.05 was considered statistically significant.

## Author Contributions

B.G., Q.J., and H.Y. conceived and designed the work; X.S. obtained the data; X.S., P.Z., Z.F., J.L., X.C., N.L., W.G., Y.H., Y.M., Y.Z., H.W., D.F., C.W., and W.W. performed in vivo studies; X.S., P.Z., J.L., Z.F., N.L., T.S., and Y.S. performed in vitro studies; W.G., J.Q., W.Y., and D.C. collected clinical samples; X.S. wrote the paper; B.G., Q.J., and H.Y. revised the paper.

## Conflicts of Interest

The authors declare no conflicts of interest.

## Supporting information




**Supporting File**: advs74535‐sup‐0001‐SuppMat.docx.

## Data Availability

The data that support the findings of this study are available in the supplementary material of this article. The RNA‐seq data have been deposited in the OMIX, China National Center for Bioinformation/Beijing Institute of Genomics, Chinese Academy of Sciences (https://ngdc.cncb.ac.cn/omix: Accession No.OMIX014987).
