# Supplementary material for: Atrophic Skeletal Muscle‐Derived Extracellular Vesicles Transfer miR‐125a‐5p to Inhibit Bone Formation in Osteoporosis during Aging
Source: Adv Sci (Weinh). 2026 Feb 26;13(25):e15362. doi: 10.1002/advs.202515362 (PMC13137847; doi:10.1002/advs.202515362)
Supplement: Supplementary file 1 — Supporting File: advs74535‐sup‐0001‐SuppMat.docx. [file ADVS-13-e15362-s001.docx]

**Supporting Information**

**Atrophic Skeletal Muscle-Derived Extracellular Vesicles Transfer miR-125a-5p to Inhibit Bone Formation in Osteoporosis During Aging**

Xiaoyan Shao^1,5,7^, Pan Zhang^1,2,3,4,7^, Zhidan Fan^5,7^, Jiaquan Lin^1,6,7^, Xiang Chen^1,2,3,4^, Na Liu^1,2,3,4^, Wang Gong^1,2,3,4^, Yi He^1,2,3,4^, Yining Zhou^1,2,3,4^, Tianshu Shi^1,2,3,4^, Yong Shi^1,2,3,4^, Yuze Ma^1,2,3,4^, Wentian Gao^1,2,3,4^, Haosheng Wang^1,2,3,4^, Depeng Fang^1,2,3,4^, Chengzhi Wang^1,2,3,4^, Wenshu Wu^1,2,3,4^, Wenjin Yan^1,2,3,4^, Jianghui Qin^1,2,3,4^, Dongyang Chen^1,2,3,4^, Haiguo Yu^5^*, Qing Jiang^1,2,3,4^*, Baosheng Guo^1,2,3,4,8^*


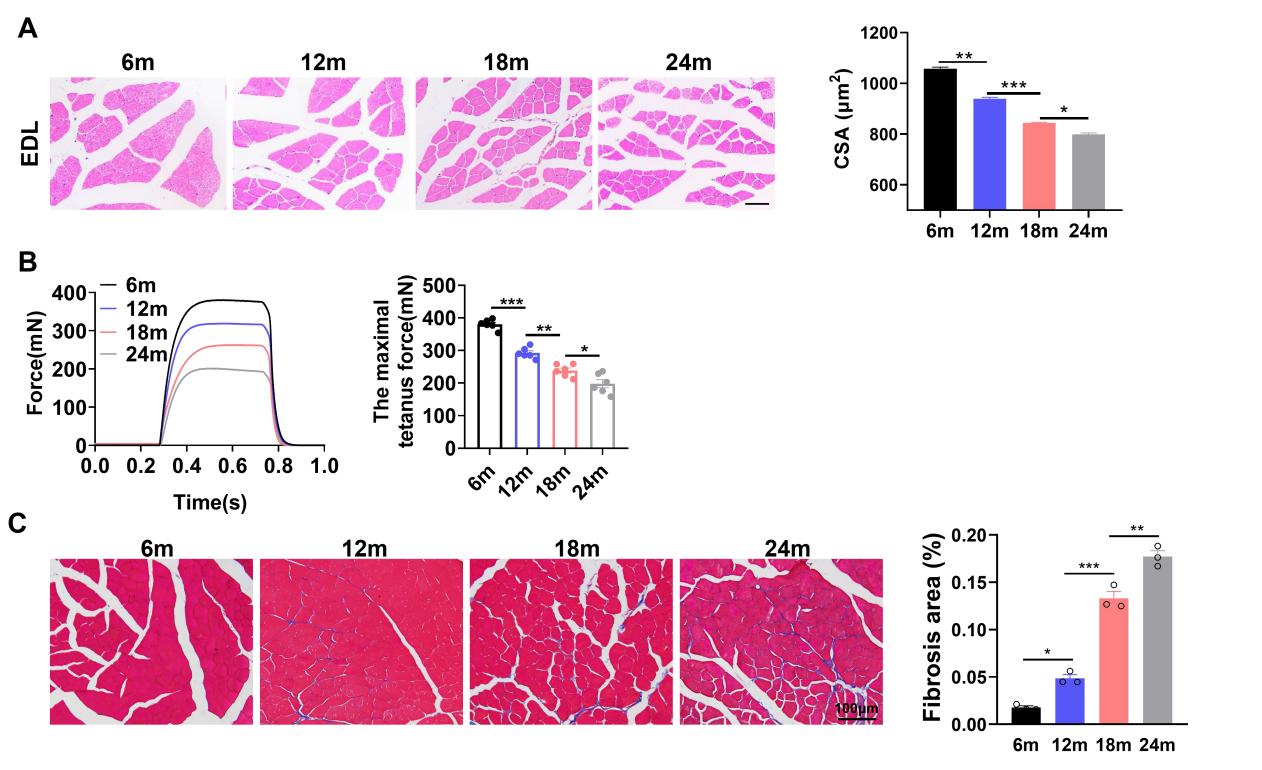


**Figure S1.** **Impaired bone formation accompanies muscle atrophy during aging in mice. (A)** Representative H&E staining of cross-sections from the mid-belly of extensor digitorum longus (EDL) muscles and quantification of muscle fiber cross-sectional area (CSA). *n = 6*. Scale bar, 100 μm. **(B)** Representative tetanic force curve of EDL muscles from 6-, 12-, 18-, and 24-month-old mice and quantification of maximal specific force during tetanic stimulation. *n = 6*. **(C)** Representative Masson’s trichrome staining of cross-sections from tibialis anterior (TA) muscles, with quantification of fibrosis area. *n = 3*. Scale bar, 100 μm. All data are presented as mean ± SEM. *P* values were determined by one-way ANOVA followed by Tukey’s multiple comparisons test **(A-C)**. **P*<0.05, ***P*<0.01, ****P*<0.001.

**
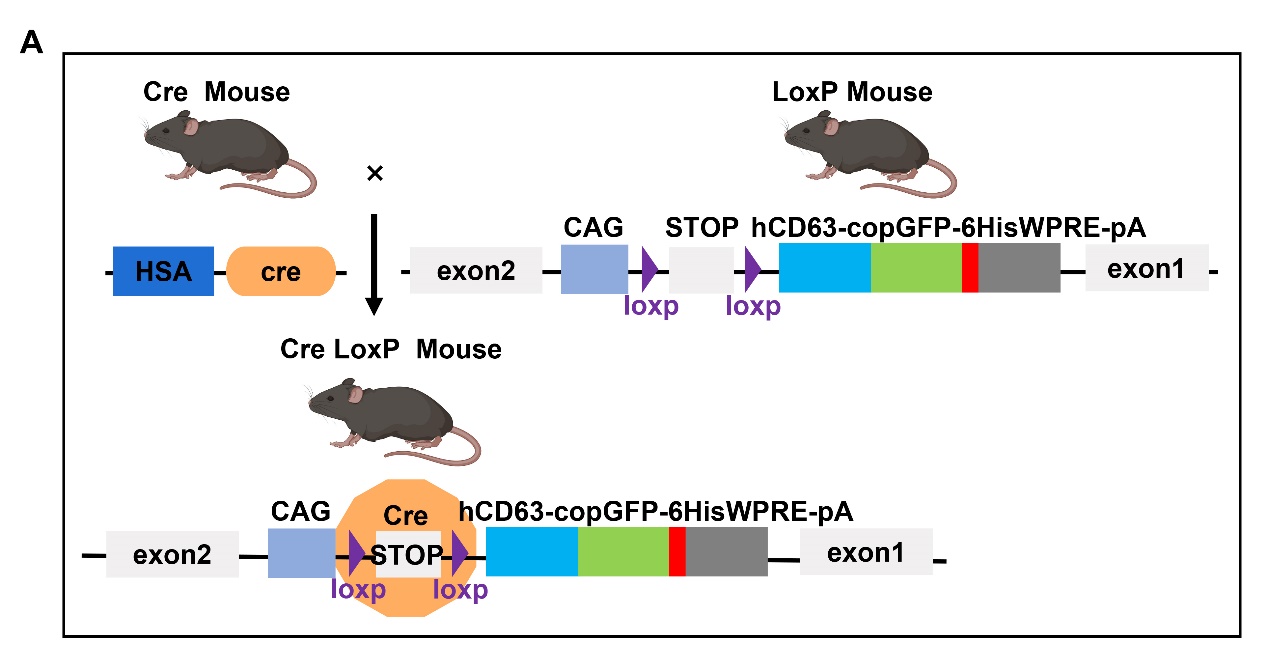
**

**Figure S2. Schematic diagram illustrating the generation of muscle-specific eGFP-labeled EV reporter mice. (A)** Strategy for generating muscle-specific eGFP-EVs reporter (*HSA^Cre^; Cd63 ^(loxp-eGFP)^*) mice. The CD63-copGFP-6xHis cassette was inserted into the Rosa26 locus, within the intron between endogenous exons 1 and 2, to generate exosome reporter *CD63-GFP^f/f^* mice. copGFP: GFP cloned from copepod *Pontellina plumata*.


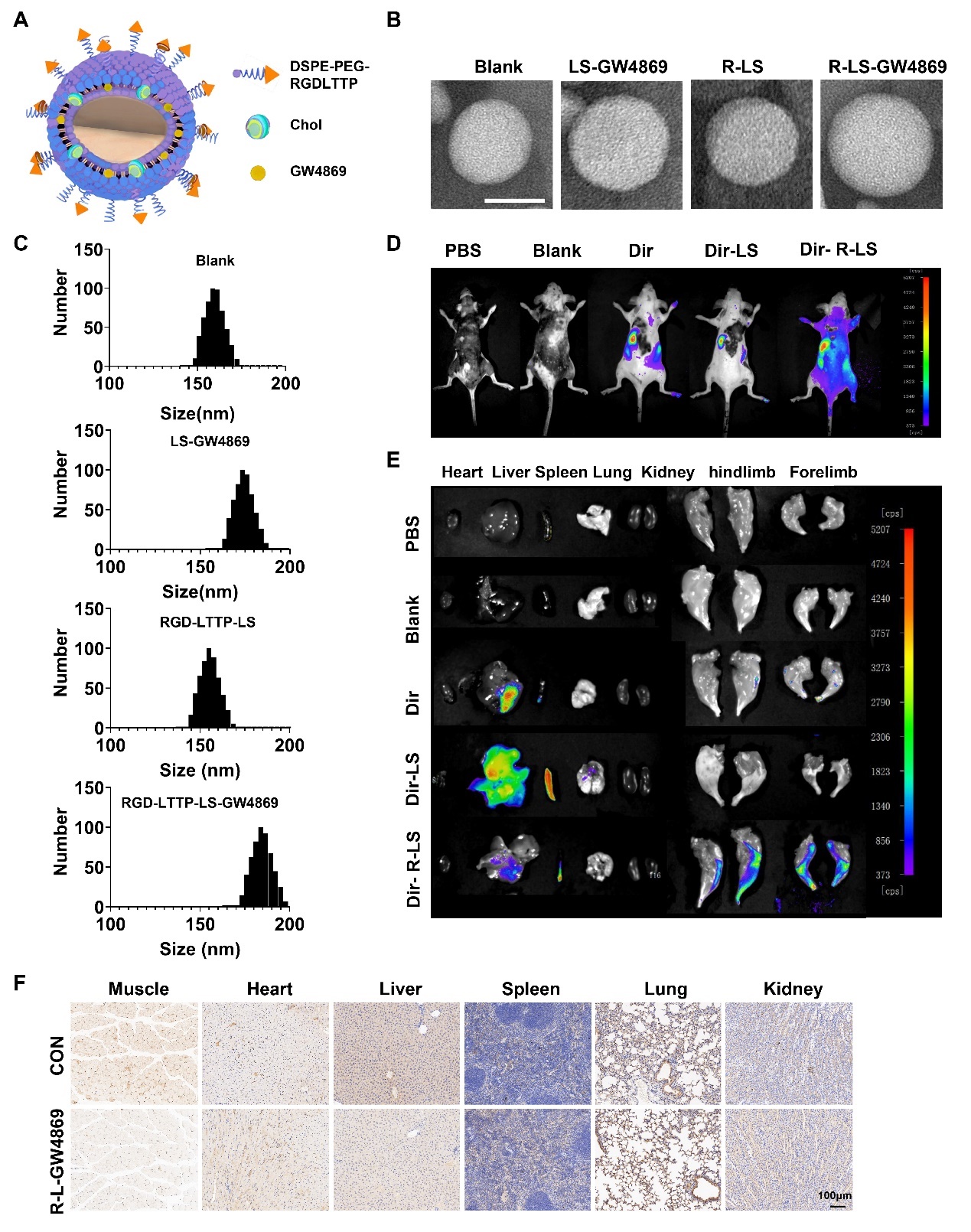


**Figure S3. Preparation, characterization, and tissue distribution of RGDLTTP peptide–modified liposomes encapsulating GW4869 (R-LS-GW4869) and analysis of nSMase2 expression. (A)** Schematic diagram illustrating the development of the R-LS-GW4869 complex. **(B)** Representative TEM images of R-LS-GW4869. **(C)** Particle size distribution of R-LS-GW4869 by dynamic light scattering (DLS). **(D)** Fluorescence imaging and intensity analysis using the IVIS Spectrum system in mice 2 days after intravenous injection with PBS, Dir, Dir-LS, or Dir-RGDLTTP-LS. **(E)** Fluorescence intensity in major organs, including heart, liver, spleen, lung, kidney, hindlimb, and forelimb, 2 days after intravenous injection with PBS, Dir, Dir-LS, or Dir-RGDLTTP-LS. **(F)** Representative immunohistochemistry staining of nSMase2. Scale bar, 100 μm.


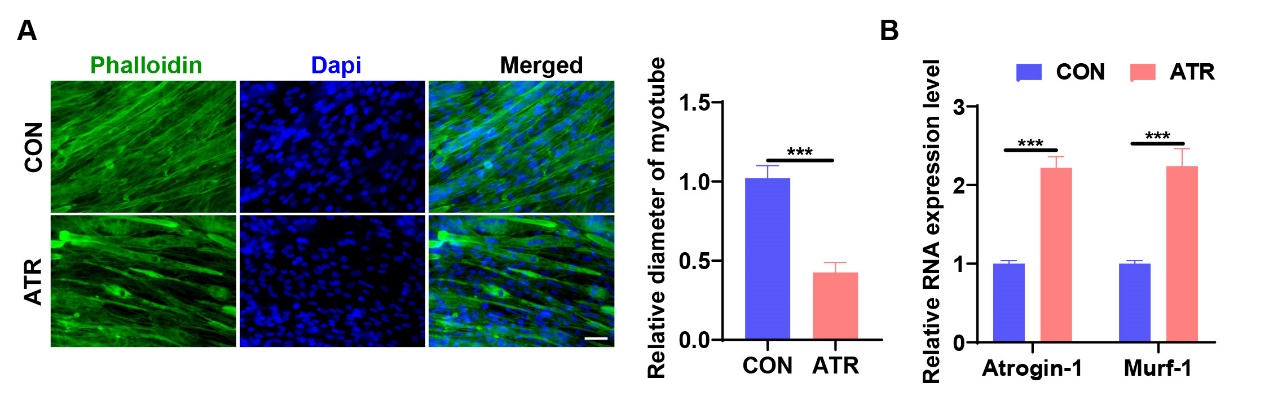


**Figure S4. The starvation-induced atrophic myotube model *in vitro*.** **(A)** Left: Representative immunofluorescence images of atrophic C2C12 myotubes following 7-hour starvation in Earle's Balanced Salt Solution (EBSS). Scale bar, 100 μm. Right: Semi-quantitative analysis of myotube diameters after 7-hour EBSS incubation. *n = 3*. **(B)** qPCR analysis of mRNA levels of muscle atrophy-related genes (*Atrogin-1 and Murf-1*) in C2C12 myotubes after 7-hour EBSS starvation. *n = 3*. All data are presented as mean ± SEM. *P* values were determined by unpaired two-tailed Student’s t test **(A-B)**. ****P*<0.001.


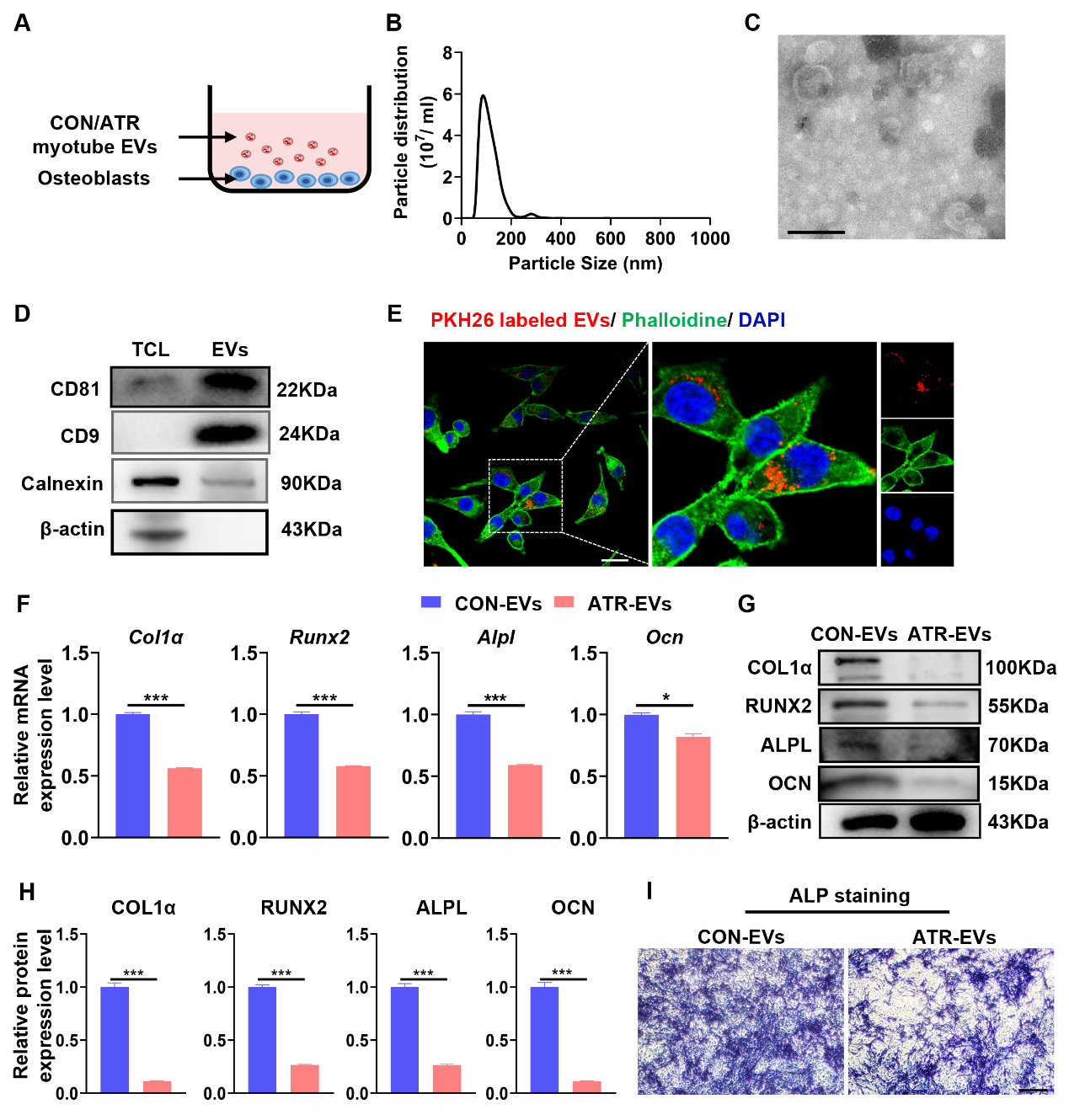


**Figure S5. Atrophic myotube derived EVs inhibit osteogenic differentiation *in vitro.* (A)** Schematic diagram illustrating the experimental design of osteoblasts incubated with CON-EVs or ATR-EVs. **(B)** NTA showing particle size distribution and concentration of the collected myotube EVs. Most detected particles were approximately 100 nm in diameter. **(C)** TEM images showing representative morphology of the collected EVs. Scale bars,100nm. **(D)** Western blot analysis confirming expression of canonical extracellular vesicle markers in the collected myotube EVs. **(E)** Representative immunofluorescence images of preosteoblasts after 24-hour incubation with PKH26-labeled myotube EVs. PKH26 (red) labels EVs, phalloidin (green) stains the cytoskeleton, and nuclei were co-stained with DAPI (blue). Scale bars, 25 μm. **(F)** qPCR analysis of osteogenic differentiation-related gene expression in preosteoblasts during differentiation after incubation with CON-EVs or ATR-EVs. *n = 3*. **(G, H)** Western blot analysis and quantification of osteogenic differentiation-related protein expression in preosteoblasts following incubation with CON-EVs or ATR-EVs. *n = 3*. **(I)** ALP staining of preosteoblasts after 7-day incubation with CON-EVs or ATR-EVs. Scale bars, 500um. *n = 3*. All data are presented as mean ± SEM. *P* values were determined by unpaired two-tailed Student’s t test **(A-B)**. **P*<0.05, ****P*<0.001.


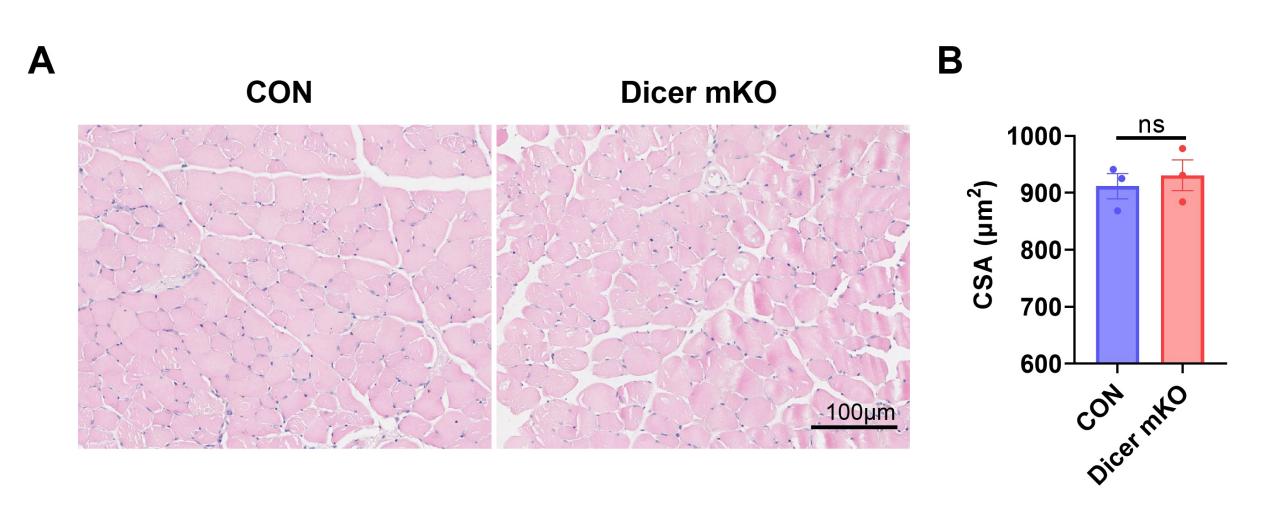


**Figure S6. Muscle phenotype of *Dicer* mKO mice.**

(A, B) Representative H&E staining of mid-belly cross-sections of TA muscles and quantification of muscle fiber CSA. n = 3. Scale bar, 100 μm. All data are presented as mean ± SEM. *P* values were determined by unpaired two-tailed Student’s t test **(B)**. ns, not significant.


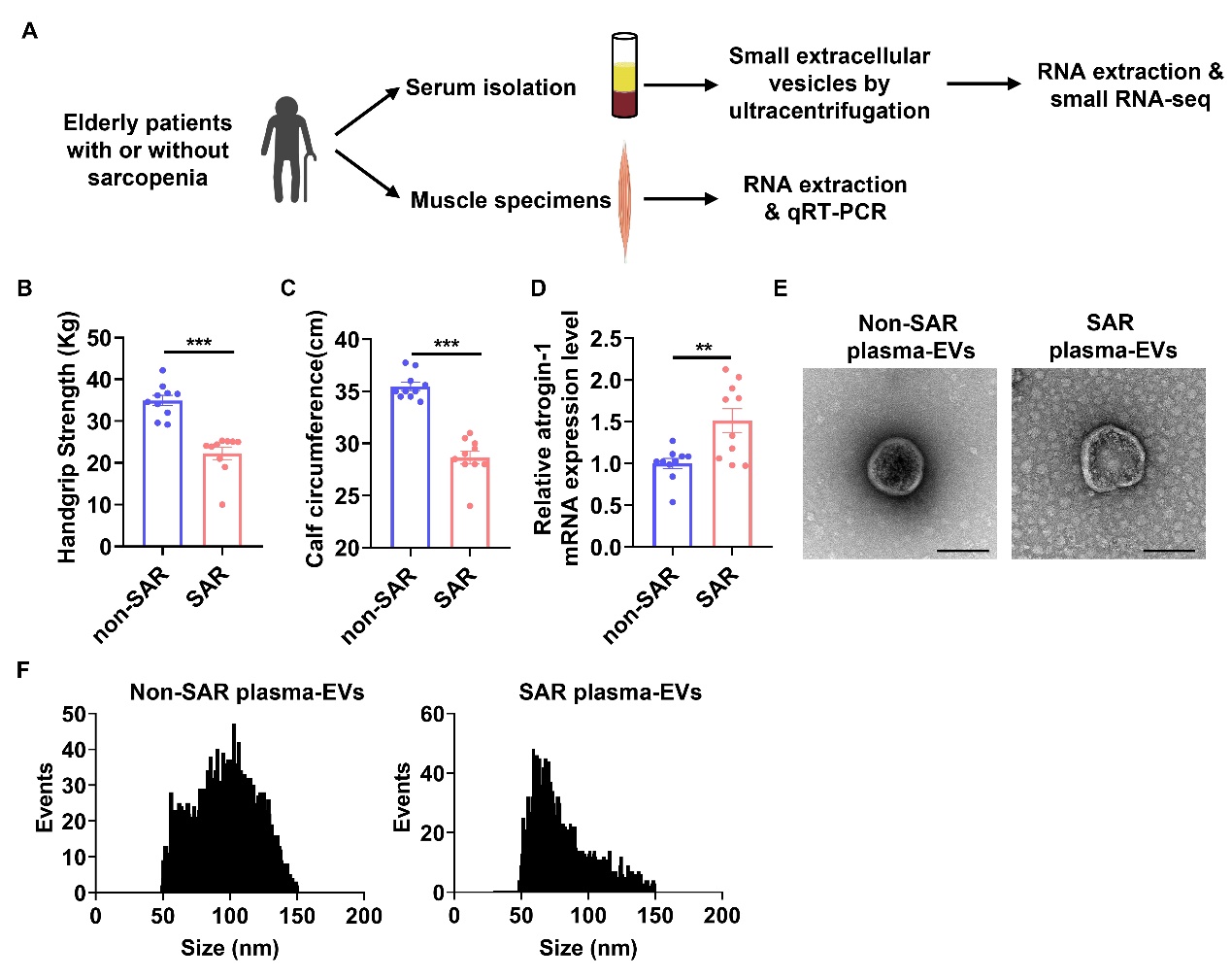


**Figure S7. Schematic diagram illustrating the experimental design for deep sequencing of small RNAs in serum EVs from sarcopenia patients.**

**(A)** Schematic overview of the experimental design. Serum and muscle samples were collected from elderly male subjects (age range: 61–79 years) with or without sarcopenia. **(B)** Handgrip strength of sarcopenia patients and non-sarcopenic subjects. *n = 10*. **(C)** Calf circumference of sarcopenia patients and non-sarcopenic subjects. *n = 10*. **(D)** qPCR analysis of Atrogin-1 mRNA levels in muscle tissues from sarcopenia patients and non-sarcopenic subjects. *n = 10*. **(E)** NTA showing particle size/concentration and particle size/relative intensity of collected serum EVs. Most detected particles were approximately 100 nm in diameter. **(F)** Representative TEM images of collected serum EVs. Scale bars, 100 nm. All data are presented as mean ± SEM. *P* values were determined by unpaired two-tailed Student’s t test **(B-D)**. ***P*<0.01, ****P*<0.001.


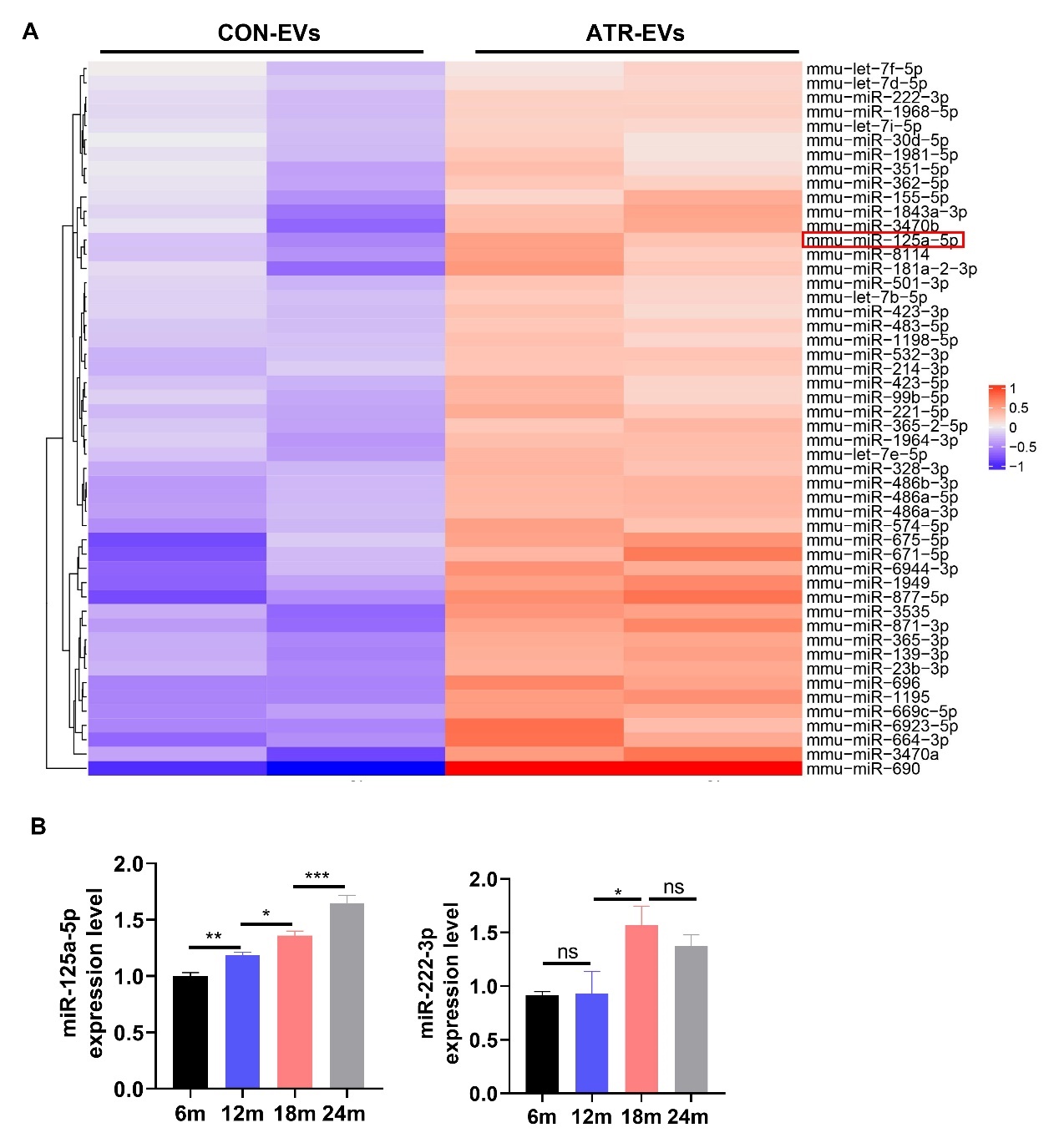


**Figure S8. miR-125a-5p is enriched in ATR-EVs and its expression in mouse skeletal muscle progressively increases with age. (A)** Heatmap showing differentially upregulated miRNAs between CON-EVs and ATR-EVs. **(B) q**PCR analysis of miR-125a-5p and miR-222-3p expression levels in skeletal muscle tissues from mice of various ages. *n = 5*. All data are presented as mean ± SEM. *P* values were determined by one-way ANOVA followed by Tukey’s multiple comparisons test **(B)**. **P*<0.05, ***P*<0.01, ****P*<0.001; ns, not significant.

**
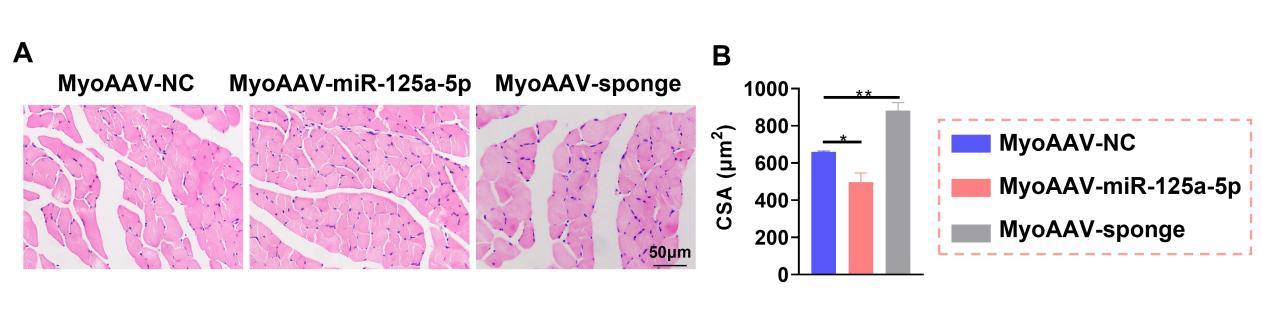
**

**Figure S9. Gain- and loss-of-function studies demonstrate that miR-125a-5p in skeletal muscle contributes to age-associated muscle atrophy. (A)** Representative H&E staining of cross-sections from the mid-belly of gastrocnemius (GAS) muscles. Scale bar, 50 μm. **(B)** Quantification of muscle fiber CSA. *n = 3.* All data are presented as mean ± SEM. *P* values were determined by one-way ANOVA followed by Tukey’s multiple comparisons test **(B)**. **P*<0.05, ***P*<0.01.

**
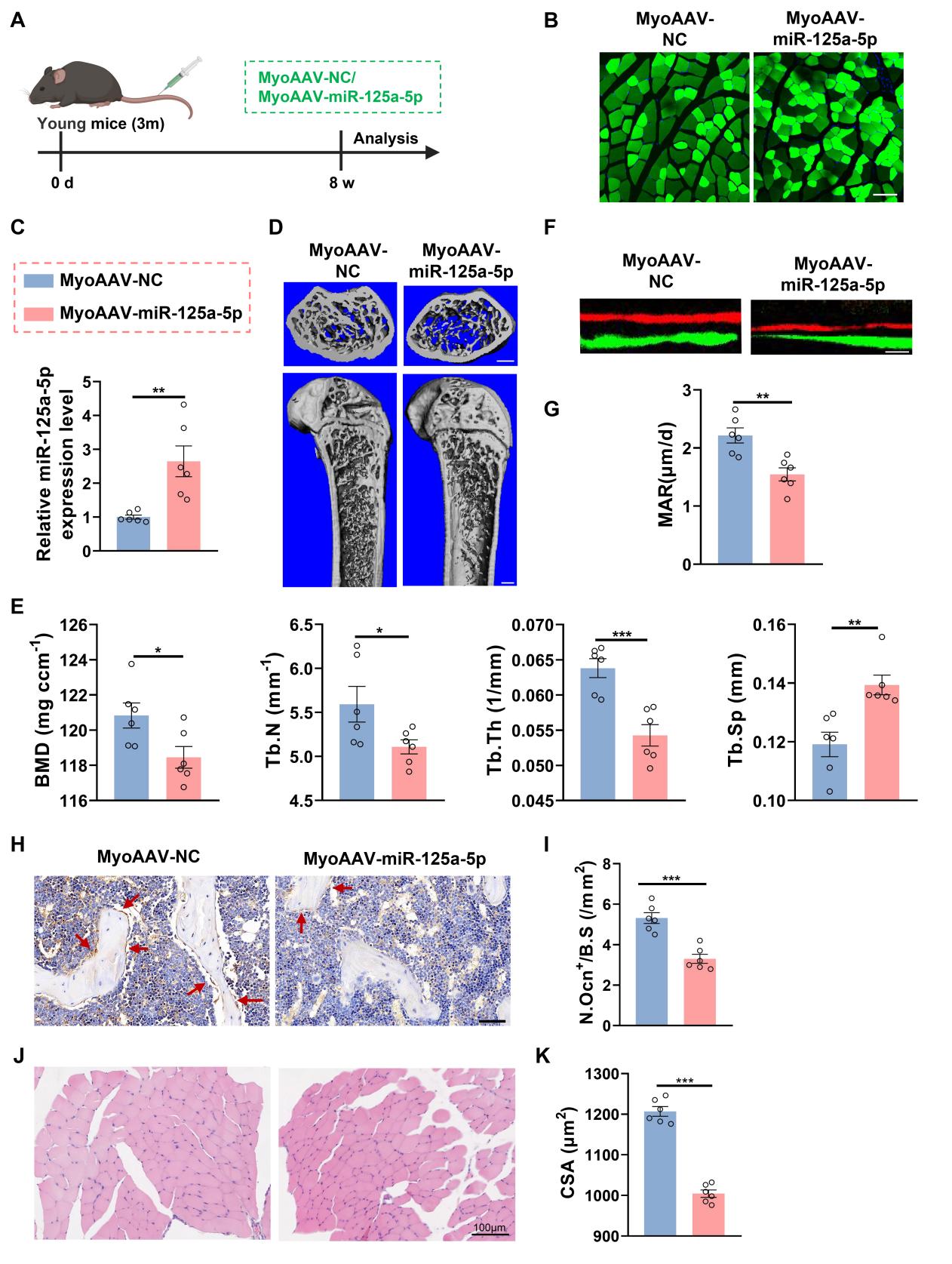
**

**Figure S10. Skeletal muscle miR-125a-5p overexpression inhibits bone formation and induces muscle atrophy in young mice.** **(A)** Schematic illustration of the experimental design. Young mice (3-month-old) were intravenously injected with recombinant MyoAAV-NC, MyoAAV-miR-125a-5p. **(B)** Representative fluorescent images of skeletal muscle sections transduced with the indicated MyoAAV vectors. **(C)** qPCR analysis of miR-125a-5p expression in skeletal muscles of mice transduced with the indicated MyoAAV vectors. *n = 6*. **(D)** Representative micro-CT reconstructed images of femur. Scale bars, 500μm. **(E)** Micro-CT analysis of BMD, BV/TV, Tb.N, Tb.Th and Tb.Sp in the distal femoral metaphysis. *n = 6*. **(F)** Representative double labelling with calcein green and xylenol orange in the distal femoral metaphysis. Scale bars, 25μm. **(G)** Bone histomorphometric analysis of MAR in the distal femoral metaphysis. n = 6. **(H)** Representative immunohistochemistry staining of OCN in the distal femur. Scale bar, 50 μm. **(I)** Quantification of Ob.S/BS at the distal femoral metaphysis. *n = 6*. All data are presented as mean ± SEM. **(J)** Representative H&E staining of cross-sections from the mid-belly of TA muscles. Scale bar, 50 μm. **(K)** Quantification of muscle fiber CSA. *n = 3.* All data are presented as mean ± SEM. *P* values were determined by unpaired two-tailed Student’s t test **(C, E, G, I, K)**. **P*<0.05, ***P*<0.01, ****P*<0.001.

**
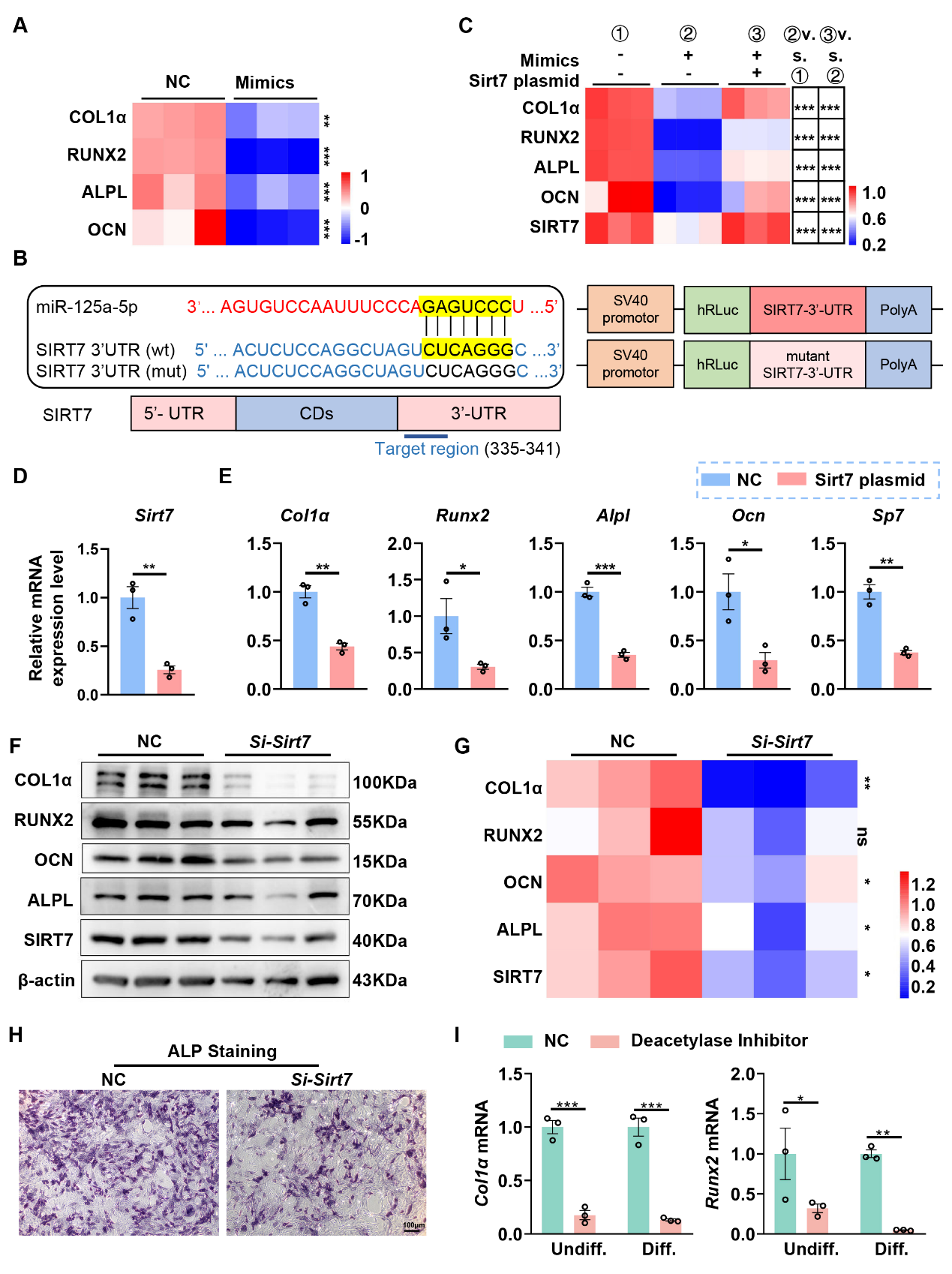
**

**Figure S11. *Sirt7* knockdown inhibits osteogenic differentiation *in vitro* by reducing *Sp7* deacetylation.** **(A**) Western blot and quantification analysis of osteogenic differentiation-related proteins in preosteoblasts transfected with miR-125a-5p mimics or NC. *n = 3*. **(B)** Schematic diagram of the psiCHECK™-2 vector construct. The predicted miR-125a-5p binding sequence and its mutated version (mut) in the 3′-UTR of SIRT7 are highlighted.  **(C)** Western blot and quantification analysis of SIRT7 and osteogenic differentiation-related proteins in preosteoblasts transfected with NC, miR-125a-5p mimics, or miR-125a-5p mimics plus Sirt7 plasmid. *n = 3*. **(D)** qPCR analysis of *Sirt7* mRNA expression in preosteoblasts transfected with NC or *si-Sirt7*. *n = 3*. **(E)** qPCR analysis of osteogenic differentiation-related genes in preosteoblasts transfected with NC or *si-Sirt7*. *n = 3*. **(F, G)** Western blot analysis and qualitification of SIRT7 and osteogenic differentiation-related proteins in preosteoblasts transfected with NC or *si-Sirt7*. *n = 3*. **(H)** ALP staining of preosteoblasts transfected with NC or *si-Sirt7*. Scale bars, 500um. **(I) q**PCR analysis of osteogenic differentiation-related genes in preosteoblasts treated with deacetylase inhibitor. *n = 3*. All data are presented as mean ± SEM. *P* values were determined by unpaired two-tailed Student’s t test **(A, D, E, G, I,)**, or **(C)**. **P*<0.05, ***P*<0.01, ****P*<0.001; ns, not significant.

**Supplementary Table 1. Primer sequences used for genotyping of genetic mice**

| *Gene* | Forward primer sequence (5’-3’) | Reverse primer sequence (5’-3’) |
| --- | --- | --- |
| *Dicer1* | CTTAGATGAGGACTTCTGGGGAGAT | AGAGCAAGTCAATCCACCAACAA |
| *Cd63 P1/2* | TCAGATTCTTTTATAGGGGACACA | TAAAGGCCACTCAATGCTCACTAA |
| *Cd63 P3/4* | TGGGGGCTGGCTGAGGAAAAATGT | TTCTCGTAGCCGCTGGGGTAGGTG |
| *Hsa*-cre | GCATGGTGGAGATCTTTGA | GCTTCTGTCCGTTTGCCGGTCG |

**Supplementary Table 2. Characters of patients**

|  | Non-sarcopenia patients (n=10) | Sarcopenia patients (n=10) | *P* value |
| --- | --- | --- | --- |
| Age | 70 ± 2.11 | 72 ± 4.78 | *P*<0.001 |
| Gender | Male | Male |  |
| Calf circumference | 34.5 ± 1.26 | 28.6 ± 1.95 | *P*<0.001 |
| Grip strength | 34.9 ± 3.97 | 22.3 ± 4.79 | *P*<0.001 |

Data are presented as mean ± SEM. Independent t test for continuous variables and chi-square for categorical values.

**Supplementary Table 3. *si-Sirt7* primer sequences**

| *Gene* | Forward primer sequence (5’-3’) | Reverse primer sequence (5’-3’) |
| --- | --- | --- |
| *si-Sirt7 (m)* | GAAGUGCCAGGCACUUGGUUGUCU | UAGACAACCAAGUGCCUGGCACUUC |

m-Mouse

**Supplementary Table 4. Primer sequences used for qRT-PCR**

| *Gene* | Forward primer sequence (5’-3’) | Reverse primer sequence (5’-3’) |  |
| --- | --- | --- | --- |
| *Atrogin-1(m)* | CAGCTTCGTGAGCGACCTC | GGCAGTCGAGAAGTCCAGTC |  |
| *Atrogin-1(h)* | GCCTTTGTGCCTACAACTGAA | CTGCCCTTTGTCTGACAGAAT |  |
| *Murf-1 (m)* | GTGTGAGGTGCCTACTTGCTC | GCTCAGTCTTCTGTCCTTGGA |  |
| *Gapdh (m)* | AGGTCGGTGTGAACGGATTTG | TGTAGACCATGTAGTTGAGGTCA |  |
| *Gapdh (h)* | CGGAGTCAACGGATTTGGTCGTAT | AGCCTTCTCCATGGTGGTGAAGAC |  |
| *Dicer1(m)* | GGTCCTTTCTTTGGACTGCCA | GCGATGAACGTCTTCCCTGA |  |
| *Col1(m)* | GCTCCTCTTAGGGGCCACT | CCACGTCTCACCATTGGGG | |
| *Runx2(m)* | ATGCTTCATTCGCCTCACAAA | GCACTCACTGACTCGGTTGG |  |
| *Alp(m)* | CCAACTCTTTTGTGCCAGAGA | GGCTACATTGGTGTTGAGCTTTT |  |
| *Ocn(m)* | GGACCATCTTTCTGCTCACTCTG | GTTCACTACCTTATTGCCCTCCTG |  |
| *Sirt7(m)* | AGCATCACCCGTTTGCATGA | GGCAGTACGCTCAGTCACAT |  |
| *Mapk14(m)* | TTTGCTCAGTACCACGACCC | GTGGTGGCACAAAGCTGATG |  |
| *Sp7(m)* | TTCGCATCTGAAAGCCCACT | TGCGCTGATGTTTGCTCAAG |  |

m-Mouse; h-Human

**Supplementary Table 5. Primers for CUT$RUN**

| *Gene* | Forward primer sequence (5’-3’) | Reverse primer sequence (5’-3’) |
| --- | --- | --- |
| *Sp7(m)* | GCCACCCATTGCCAGTAATC | CTGCCCAGTGTCTGTACCTG |

m-Mouse

**Supplementary Table 6. Antibodies used for WB or IHC/IF**

| Primary antibodies | Company | Catalog No. | Use | Concentration |
| --- | --- | --- | --- | --- |
| RAB27A | ABclonal | A23993 | IHC  WB | 1:200  1:1000 |
| CD9 | Proteintech | 20597-1-AP | WB | 1:1000 |
| CD81 | Proteintech | 66866-1-lg | WB | 1:1000 |
| CD63 | Abcam | ab252919 | WB  IF | 1:1000  1:200 |
| TSG101 | Abcam | ab125011 | WB | 1:1000 |
| OCN | ABclonal | A6205 | WB | 1:1000 |
| OCN | BIOSS | bs0470R | IF、IHC | 1:200 |
| COL1α1 | ABclonal | A1352 | WB | 1:1000 |
| RUNX2 | Zen-bio | R25634 | IF | 1:200 |
| ALPL | ABclonal | A0514 | WB | 1:1000 |
| SIRT7 | Proteintech | 12994-1-AP | WB | 1:1000 |
| Calnexin | Proteintech | 10427020AP | WB | 1:1000 |
| nSMase2 | Signalway | C01004 | IHC | 1:200 |
| IgG | HUABIO | HA722127 | CUT&RUN | 1 μg |
| SIRT7 | HUABIO | HA723075 | CUT&RUN | 1 μg |
| β-actin | ABclonal | AC026 | WB | 1:1000 |
| α-tubulin | ABclonal | AC012 | WB | 1:1000 |
